# Unbiased proteomic analysis of extracellular vesicles secreted by senescent human vascular smooth muscle cells reveals their ability to modulate immune cell functions

**DOI:** 10.1007/s11357-022-00625-0

**Published:** 2022-07-28

**Authors:** Agata Głuchowska, Dominik Cysewski, Monika Baj-Krzyworzeka, Rafał Szatanek, Kazimierz Węglarczyk, Paulina Podszywałow-Bartnicka, Piotr Sunderland, Ewa Kozłowska, Małgorzata A. Śliwińska, Michał Dąbrowski, Ewa Sikora, Grażyna Mosieniak

**Affiliations:** 1 Laboratory of Molecular Bases of Aging, Nencki Institute of Experimental Biology, Polish Academy of Sciences, Pasteura 3 St., 02-093 Warsaw, Poland; 2 Mass Spectrometry Laboratory, Institute of Biochemistry and Biophysics, Polish Academy of Sciences, Warsaw, Poland; 3 Clinical Research Centre, Medical University of Bialystok, Białystok, Poland; 4 Department of Clinical Immunology, Institute of Pediatrics, Jagiellonian University Medical College, Krakow, Poland; 5 Laboratory of Flow Cytometry, Nencki Institute of Experimental Biology, Polish Academy of Sciences, Warsaw, Poland; 6 Department of Immunology, Institute of Functional Biology and Ecology, Faculty of Biology University of Warsaw, Warsaw, Poland; 7 Laboratory of Imaging Tissue Structure and Function, Nencki Institute of Experimental Biology Polish Academy of Sciences, Warsaw, Poland; 8 Laboratory of Bioinformatics, Neurobiology Centre, Nencki Institute of Experimental Biology of Polish Academy of Sciences, Warsaw, Poland

**Keywords:** Senescence, Human smooth muscle cells, Extracellular vesicles, Exosomes, Secretome, Immune cells

## Abstract

Atherosclerosis, a common age-related disease, is characterized by intense immunological activity. Atherosclerotic plaque is composed of endothelial cells, vascular smooth muscle cells (VSMCs), lipids and immune cells infiltrating from the blood. During progression of the disease, VSMCs undergo senescence within the plaque and secrete SASP (senescence-associated secretory phenotype) factors that can actively modulate plaque microenvironment. We demonstrated that senescent VSMCs secrete increased number of extracellular vesicles (senEVs). Based on unbiased proteomic analysis of VMSC-derived EVs and of the soluble fraction of SASP (sSASP), more than 900 proteins were identified in each of SASP compartments. Comparison of the composition of VMSC-derived EVs with the SASP atlas revealed several proteins, including Serpin Family F Member 1 (SERPINF1) and Thrombospondin 1 (THBS1), as commonly upregulated components of EVs secreted by senescent VSMCs and fibroblasts. Among soluble SASP factors, only Growth Differentiation Factor 15 (GDF15) was universally increased in the secretome of senescent VSMCs, fibroblasts, and epithelial cells. Bioinformatics analysis of EV proteins distinguished functionally organized protein networks involved in immune cell function regulation. Accordingly, EVs released by senescent VSMCs induced secretion of IL-17, INFγ, and IL-10 by T cells and of TNFα produced by monocytes. Moreover senEVs influenced differentiation of monocytes favoring mix M1/M2 polarization with proinflammatory characteristics. Altogether, our studies provide a complex, unbiased analysis of VSMC SASP and prove that EVs derived from senescent VSMCs influence the cytokine milieu by modulating immune cell activity. Our results strengthen the role of senescent cells as an important inducer of inflammation in atherosclerosis.

**Supplementary Information:**

The online version contains supplementary material available at 10.1007/s11357-022-00625-0.

## Introduction

Atherosclerosis represents an age-related disease, which remains one of the leading causes of death in developed countries. It begins with a local deposition of lipids in the innermost part of the artery — the intima. Thereafter, a number of immune cells are attracted and infiltrate into the artery wall where, together with endothelia and smooth muscle cells, they form an atherosclerotic plaque. Thus, atherosclerosis is considered as an inflammatory disease, characterized by intense immunological activity [1]. One of the main risk factors for atherosclerosis is aging. It was demonstrated that senescent cells accumulate within atherosclerotic plaques [2]. Accordingly, vascular smooth muscle cells (VSMCs) derived from the lesion demonstrate a phenotype characteristic for senescent cells: diminished proliferative potential, shorter telomeres, increased level of DNA damage, and upregulation of SA-β-gal activity [3]. Accumulation of senescent VSMCs within the area of plaque formation promotes its instability due to lack of proliferation and impaired production of extracellular matrix, both leading to the weakening of the fibrous cap [4]. Moreover, elimination of senescent cells in the atherosclerosis-prone low-density lipoprotein receptor-deficient (Ldlr-/-) mice slowed down the disease progression [5]. One of the most important features of senescent cells, that has the biggest impact on tissue microenvironment, is their ability to secrete a number of cytokines, chemokines, matrix metalloproteinases, and growth factors, collectively known as the senescence-associated secretory phenotype (SASP) [6]. Apart from soluble factors, senescent cells also secrete an increased number of extracellular vesicles (EVs), which have begun to be studied only recently [7]. EVs are small membranous vesicles that can be categorized, based on their size and origin, into exosomes, microvesicles, and apoptotic bodies. EVs play crucial role as carriers of proteins, different types of RNA and DNA, lipids, and metabolites that take part in intercellular communication [8]. They were shown to participate in many physiological but also pathological processes.

Studies performed over the last decade proved that EVs affect atherosclerosis progression at different stages by several distinct mechanisms [9]. Increased concentrations of EVs have been found in plasma and atherosclerotic plaque of patients [10, 11]. However, the role of EVs derived from senescent cells in atherosclerosis, particularly in the context of plaque development, remains unknown. The functioning of immune cells within an atherosclerotic plaque is modulated by a local milieu. Thus, senescent cells present in the lesion can influence plaque formation by a non-cell autonomous mechanism. Indeed, it has been demonstrated recently that SASP factors secreted by senescent VSMCs promote recruitment of monocytes, induce expression of adhesion receptors on endothelial cells, and activate adjacent normal VSMCs, promoting inflammation in the plaque [12]. However, the role of EVs in this context has not been studied. Thus, we have undertaken research aimed at investigating the role of senescent cell-derived EVs in immune cell activity.

To this end, we isolated and characterized the EVs secreted by human VSMCs undergoing senescence. We performed an unbiased quantitative proteomic analysis of both soluble and insoluble SASP components. Moreover, we analyzed the influence of EVs derived from senescent and non-senescent cells on T lymphocytes and monocytes. Altogether our studies revealed that EVs produced by senescent VSMCs strengthen the inflammatory response of the immune cells, which could have a tremendous significance for atherosclerotic plaque development.

## Materials and methods

### Culture of vascular smooth muscle cells

Primary human vascular smooth muscle cells (VSMCs) were purchased from Lonza or from ATCC. Cells were cultured in SmBM medium (Lonza, Switzerland) or vascular cell basal medium (ATCC, LGC, Poland) supplemented as defined by the manufacturer, and kept in humidified atmosphere (37 °C and 5% CO_2_ in the air). To obtain population of cells that underwent stress-induced premature senescence (SIPS), VSMCs were seeded at a density of 8000 cells/cm^2^, were treated with one dose of H_2_0_2_ (150 µM) 24 h later, and cultured for 7 days. Simultaneously, VSMCs were passaged every 3–4 days till they lost proliferative potential and reached the state of replicative senescence (RS). For further experiments, we used only VSMC cultures, in which the amount of SA-β-gal positive and BrdU negative cells was higher than 80%. As a control we used cells in the phase of intensive growth (passage 4–9).

### EV isolation

EVs were isolated according to a commonly used protocol with some modifications [13]. In brief, VSMC cultures (control and senescent) were washed one time with PBS and cells were incubated for 24 h in serum free medium to obtain conditioned medium (CM). After 24 h, CM was collected and centrifuged using a MPW-350R centrifuge at 1280 g at 4 °C for 10 min. Then, the supernatant was collected and centrifuged in a Beckman Coulter Optima XPN-100 Ultracentrifuge at 26.200 g at 4 °C for 40 min in a Type 45 Ti rotor (k-Factor 133). After that, the supernatant was transferred to fresh polycarbonate centrifuge bottles and centrifuged at 142,000 g at 4 °C for 100 min in a Type 45 Ti rotor. After centrifugation, the supernatant was collected and stored at − 80 °C for further analysis as EV-free medium. The obtained EV pellet was suspended in 50 µl of PBS and stored under the same conditions.

### Extracellular vesicles characterization

#### TEM visualization

Freshly isolated EVs were fixed with 2% paraformaldehyde (Sigma Aldrich,) in PBS, and 5 µl of the suspension was transferred onto formvar-coated cooper EM grids and incubated for 20 min. A 200-µl drop of ddH_2_O was placed on a parafilm and grids transferred with the sample membrane side facing down, for 1 min. Then, the grids were transferred to a 100-µl drop of 4% aqueous uranyl acetate for 10 min on ice. Finally, the grids were gently blotted from excess fluid on a Whatman filter paper and stored in appropriate grid storage boxes. EVs were visualized using transmission electron microscopy JEM 1400 (JEOL Co.)

#### Size measurement

The mean size and concentration of EVs were analyzed using NanoSight NS500 (Malvern Instruments Ltd.). For each measurement, nine 1-min videos were captured and analyzed by the in-build NanoSight Software NTA 3.2 Build 3.2.16. Before measurement, each sample was diluted 1:500 in PBS. EVs from at least 9 independent isolations from each experimental conditions, control, SIPS, and RS, cells were analyzed.

#### Western blot analysis

EVs were lysed in reducing sample buffer: 125 mM Tris–HCl (pH 6.8), 4% SDS, 20% glycerol, 100 mM DTT, and 0.2% bromophenol blue or non-reducing sample buffer (without DTT) and denaturated for 10 min at 95 °C. Total protein concentration was estimated using bicinchoninic acid (BCA) protein assay kit, and 20 µg of each sample was loaded on a gel. Alternatively, proteins isolated from EVs, that were secreted by the same number of cells, were loaded on the gel. Proteins were resolved by SDS-PAGE, transferred to nitrocellulose, and blocked in 5% non-fat powdered milk in TBS containing 0.1% Tween-20 (TBST) or in 5% BSA in TBST for 1 h and probed with antibodies overnight at 4 °C. The primary antibodies used were anti-Flotilin-1 (BD Transduction Laboratories, 1:500), anti-CD63 (Abcam 1:1000), anti-CD81 (Abcam, 1:500), anti-GM130 (Cell Signaling 1:1000), and anti-TOM20 (GeneTex, 1:500). After incubation with the horseradish peroxidase-conjugated secondary antibodies (Dako, 1:2000), protein bands were detected using X-ray film and enhanced by ECL reagent (Thermo Scientific).

#### ExoElisa

The number of CD63 expressing EVs secreted by VSMCs was analyzed using ExoElisa-Ultra Complete Kit (System Biosciences) according to the manufacturer procedure. Absorbance was measured at 450 nm using a Tecan Sunrise spectrophotometer (Tecan) and analyzed with the X-fluor 4 software. EVs from at least 8 independent isolations from each experimental conditions — control, SIPS, and RS cells — were analyzed.

### T cell isolation and activation

Human T cells were isolated from buffy coats of blood samples obtained from healthy volunteer donors, in accordance with local ethical regulations, and provided by the Domestic Blood Center in Warsaw, Poland. Isolation was performed using the RosetteSep Human T cell enrichment cocktail (StemCell Technologies,), according to the manufacturer procedure.

To activate isolated T cells, T cell Activation/Expansion Kit human (Miltenyi Biotec) was used. The Anti-biotin MACSiBead particles were loaded with CD2, CD3, and CD28 antibodies according to the manufacturer protocol and purified T cells were activated using one loaded anti-biotin particle per two T cells. T cells were seeded at a density of 1 × 10^6^ cells per 1 ml of medium and kept in humidified atmosphere.

### Uptake of VSMC-EVs by human T cells

EVs were isolated using the ultracentrifugation method as described above. After 142,000 × g ultracentrifugation, EVs were incubated with 7.5 µM CFSE (Invitrogen) for 30 min at 37 °C in PBS containing 0.5% BSA. Labeled EVs were diluted with vascular cell basal medium without FBS and ultracentrifuged for 100 min at 142,000 × g. Pelleted EVs (~ 30 µg) were resuspended in medium and incubated with activated T cells at 37 °C for 4 h. The uptake of EVs was visualized in bright-field and fluorescence (Ex 488 nm) under confocal microscopy (Leica SP8).

### Measurement of the level of T cell activation

Activation of T cells was estimated based on expression of CD25, CD69, and CD38. The measurements were performed 24 h after culturing the cells in the presence of activation beads in culture medium as described: conditioned medium (CM), EV-free medium, and fresh medium supplemented with EVs. Media were collected from the same number of control, SIPS, and RS VSMCs. Cells were fluorescently stained using Viability dye-DAPI (eBioscience), anti-Hu CD4-Alexa Fluor 700 (eBioscience), anti-Hu CD8a-PE (eBioscience), anti-Hu CD25-APC (eBioscience), anti-Hu CD69-PerCP (eBioscience), and anti-Hu CD38-PE-Cyanine 7 (eBioscience) and measured in a BD LSRFortessa flow cytometer. Data were analyzed using FlowJo software. T cells isolated from at least 9 donors were analyzed.

### Determination of cytokine secretion by T cells

The amount of cytokines secreted by T cells upon different culture conditions was measured using Cytometric Bead Array (CBA) Human Th1/Th2/Th17 Cytokine kit (BD Biosciences). 200 µl of medium was collected 72 h after activation, centrifuged at 300 × g for 5 min, and kept at -80 °C until cytokine measurement. The assay was performed according to a dedicated protocol. Cytokines IL-17a, IL-10, IL-2, IL-4, TNF, and IFN-γ were estimated using a BD LSRFortessa flow cytometer. Data were analyzed with BD FCAP Array TM 3.0 software. T cells isolated from at least 8 donors were analyzed.

### Isolation of monocytes

Monocytes were separated from mononuclear cells by counter-flow centrifugal elutriation with the JE-5.0 elutriation system equipped with a 5-ml Sanderson separation chamber (Beckman, Palo Alto CA) as described before [14].

### Differentiation of monocytes into MDM

Blood monocytes were differentiated in the presence of EVs (control, SIPS and RS) added to the culture at the ratio 1000:1 (EVs to monocytes) for 7 days. The efficiency of differentiation was evaluated as described before [14]. Monocytes isolated from at least 8 donors were analyzed .

### Isolation of monocyte subpopulations

The CD14 +  + CD16-, CD14 +  + CD16 + , and CD14 + CD16 +  + monocyte subsets were isolated from whole population of monocytes by cell sorting (FACS Aria cell sorter, BD Biosciences Immunocytometry Systems, San Jose CA) after labeling with anti- CD14 APC and anti-CD16 PE (BD Pharmingen, San Diego CA).

### Determination of cytokine secretion by monocytes

Monocyte subsets were cultured in RPMI medium with or without addition of EVs overnight at 37 °C, 5% CO_2_ in humidified atmosphere. After 18 h, culture supernatants were collected and concentration of TNF and IL-10 was measured using the Flex Set system (BD Bioscience, San Diego CA) according to the manufacturer protocol. Monocytes isolated from at least 11 donors were analyzed.

### Proteomic sample preparation and mass spectrometry analysis

Collected samples were lysed in 2% SDS, 100 mM TRIS, 50 mM TCEP, 96C for 5 min. Lysates were subjected to sonication at high amplitude with 30 s on/30 s off cycle for 30 min (Diagenode Bioruptor XL). Materials from three independent biological experiments for each condition (control, SIPS, and RS) were subjected into proteomic analysis.

Samples were then prepared based on a modified FASP protocol [15]. Briefly, supernatant was placed at a Vivacon 30 kDa filter, centrifuged, and washed 3 times with 200 µl of 8 M Urea in 100 mM NH_4_HCO_3_. Afterwards, the supernatant was reduced (DTT, RT, 30 min) and alkylated (IAA, RT, 15 min), digested overnight with trypsin (Promega), and acidified with TFA to a final concentration of 0.1%. The final peptide mixture was measured and labeled with isobaric tags. Samples were then labeled using the standard TMT10 (Thermo Fisher Scientific) protocol according to manufacturer recommendations. The labeled peptide mixture was speed-vaced to dry and then dissolved in 100 μl of 2% MeCN/0.1% TFA.

Samples were analyzed at the Laboratory of Mass Spectrometry (IBB PAS, Warsaw) using a nanoAcquity UPLC system (Waters) coupled to an Orbitrap Q Exactive mass spectrometer (Thermo Fisher Scientific). The mass spectrometer was operated in the data-dependent MS2 mode, and data were acquired in the m/z range of 300–1600. Peptides were separated by a 180-min linear gradient of 95% solution A (0.1% v/v formic acid in water) to 35% solution B (acetonitrile and 0.1% formic acid). The measurement of each sample was preceded by three washing runs to avoid cross-contamination. The final MS washing run was searched for the presence of cross-contamination between samples. The working parameters were capillary voltage 3 kV, capillary temperature 250 °C, MS1 resolving power: 70,000, AGC 10e6, MS2 resolving power 35,000 for TMT samples, AGC 5e5, collision energy 27%, loop count 12, and isolation window 1.2 m/z,

Data were searched with the MaxQuant (1.6.0.16) against the UniProt database reference proteome (human proteome, 75,004 entries). Search parameters: variable modification: oxidation (M), acetyl (N-term), minimal peptide length 7–25 aa, peptide mass tolerance 20 ppm, fragment mass tolerance 0.5 Da, digestion trypsin/specific, and FDR 1% on peptide and protein identification level.

For TMT10-plex labeled samples, reporter mass tolerance is 0.003 Da.

Results were statistically and quantitatively analyzed using the Scaffold 4 platform (Proteome Software); normalized TMT reporter intensities were used for quantitative data interpretation. Groups were compared by Whitney-Mann statistical test with Benjamin-Hochberg correction for *p*-value 0.05. Due to the nature of sample, i.e., the presence of many short proteins, identification was performed also for proteins identified with one peptide at 1% FDR confidence. Fold change (FC) was calculated at the protein level. Data were plotted in a volcano-plot format (− log10(*p*-value) vs log10FC.

For TMT10-plex labeled samples, reporter mass tolerance is 0.003 Da.

The mass spectrometry proteomics data are available via ProteomeXchange with identifier PXD030955.

Reviewer account details: Username: reviewer_pxd030955@ebi.ac.uk; Password: 6StTO0yk.

### Proteomic data visualization

Venn diagrams were constructed using the Functional Enrichment Analysis tool FunRich 3.1.3 (http://www.funrich.org/). Selected pathways for enrichment analysis were referenced from Reactome Pathways database using g:Profiler (https://biit.cs.ut.ee/gprofiler/gost). The statistical threshold for enriched pathways was Bonferroni adjusted *p*-values < 0.05. Pathway and network analysis and visualizations were performed and modified using the ClueGo package, version 2.5.8 in Cytoscape version 3.8.2 (https://cytoscape.org/).

### Statistical analysis

Statistical analysis was performed using GraphPad Prism version 8.3.1 (San Diego, California USA). Data were presented as the means ± SEM. Samples were analyzed using unpaired Student’s *t*-test or one-way ANOVA with post hoc Bonferroni’s test as indicated; with *p* < 0.05 deemed statistically significant and denoted by asterisks; *p* < 0.05, *; *p* < 0.01, **; *p* < 0.001, ***; *p* < 0.0001, ****

## Results

### senVSMCs secrete increased number of EVs that are enriched in exosomes

In order to study the role of EVs secreted by senescent VSMCs, we took advantage of two experimental setups that we described previously [16]. In the model of stress induced premature senescence (SIPS), human VSMCs were treated with a single dose of H_2_O_2_ and cultured for subsequent 7 days. In the alternative model, the cells were cultured until they lost the replication potential and underwent replicative senescence (RS). Analysis of SA-β-gal activity and Ki-67 expression confirmed that majority of cells became senescent (Fig. S1).

**Fig. 1:**
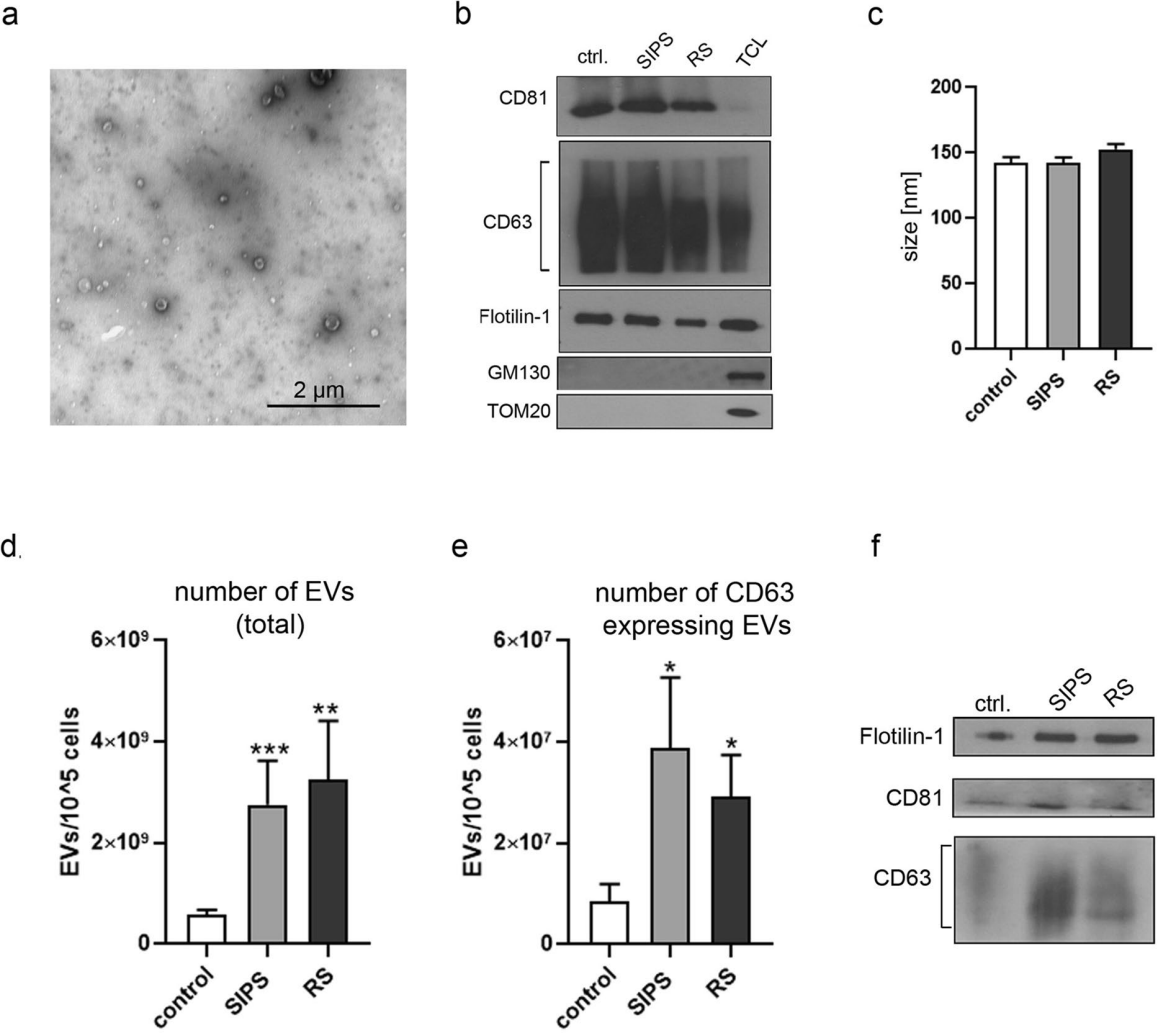
Characterization of extracellular vesicles secreted by VSMCs. **a** Isolated EVs, obtained after ultracentrifugation of conditioned medium, observed in TEM; **b** Western blot analysis of common exosome markers (CD81, CD63, and Flotilin-1) in EV fraction (20µg of each sample). The purity of isolated EVs was confirmed by lack of expression of Golgi (GM130) and mitochondrial (TOM20) markers, TLC — total cell lysate; **c** Size measurement of EVs using NanoSight nanoparticle tracking analysis (NTA). **d** The total number of EVs secreted by young and senescent cells measured by NTA; EVs from at least 9 independent isolations from each experimental conditions, control, SIPS and RS, cells were analyzed. **e** Comparison of the number of CD63 positive EVs secreted by VSMCs (ExoElisa); EVs from at least 9 independent isolations were analyzed. **f** Representative blots presenting the differences in the level of CD81, CD63, and Flotilin-1 protein in EVs secreted by 2 × 10^5^ young and senescent cells

Our previous data have shown that senescent VSMCs secrete increased amount of SASP factors, such as IL-6, IL-8, and VEGF [16]. In the current studies we analyzed the vesicular components of SASP-EVs. EVs secreted by VSMCs were isolated from non-serum cell culture medium by ultracentrifugation after 24 h of conditioning. Isolated vesicles were visualized using transmission electron microscopy (TEM) (Fig. 1a). Based on Western blot analysis of CD63, CD81, and Flotilin-1, the protein markers of exosomes, we confirmed that the isolated fraction of vesicles is enriched in exosomes. The purity of isolated EVs was also verified by the lack of *cis-*Golgi (GM130) and mitochondrial (TOM20) markers (Fig. 1b). We did not observe any differences in the size of EVs derived from non-senescent and senescent cells (Fig. 1c). However, VSMCs senescing due to SIPS or RS were shown to secrete significantly more EVs than control cells (Fig. 1d). Moreover, senEVs contained significantly more exosomes than EVs from control cells as demonstrated by ExoElisa analysis of the number of CD63 positive vesicles (Fig. 1e) and WB of exosome markers in the EV fraction secreted by the same number of cells (Fig. 1f).

### The diversity of secretomes of VSMCs undergoing H_2_O_2_-induced (SIPS) and replicative (RS) senescence revealed by unbiased proteomic analysis

**Fig. 2:**
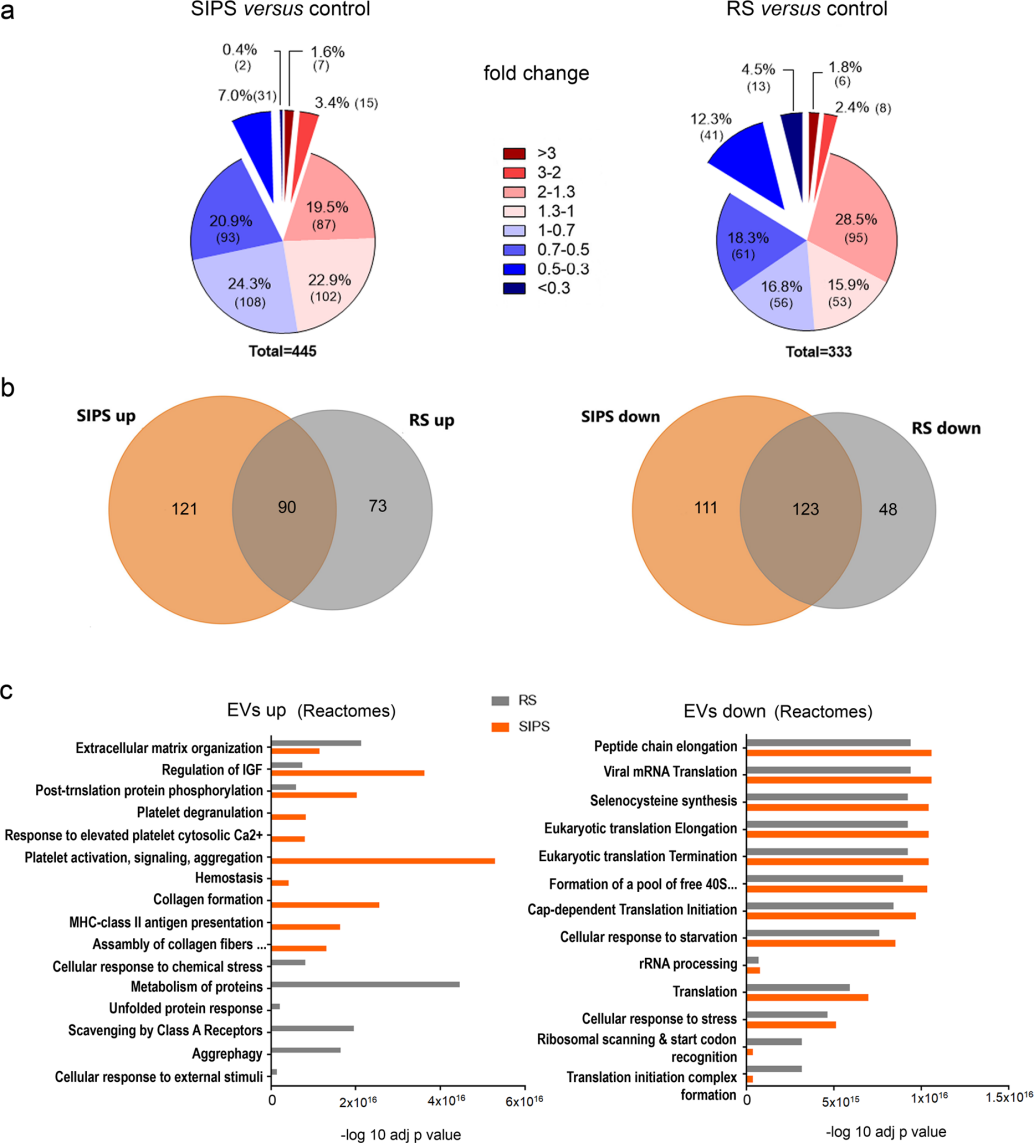
Comparative proteomic analysis of proteomes of EVs secreted by control and senescent VSMCs. **a** Pie charts presenting the distribution of fold changes (FC) in the level of proteins identified in EVs secreted by senescent cells relative to EVs from control cells (p < 0.05). **b** Venn diagrams of up and downregulated proteins identified in EVs from SIPS and RS cells that were significantly changed (*p* < 0.05); **c** Reactome pathway analysis of proteins that were upregulated (FC > 1.3) or downregulated (FC < 0.7) in EVs secreted by senescent VSMCs (SIPS and RS) comparing to EVs from young VSMCs. EVs from three independent biological experiments for each condition (control, SIPS, and RS) were subjected into proteomic analysis

To get further insight into differences and similarities between EVs secreted by non-senescent (control) and senescent cells, we performed proteomic analysis of isolated EVs using the tandem mass tag (TMT) method. We analyzed the proteome of EVs and of the soluble fraction of SASP (sSASP), i.e., proteins present in the supernatant separated from EVs by the last ultracentrifugation. A similar number of proteins were identified (975 in EVs and 875 in sSASP). We compared the relative amount of proteins present in EVs secreted by senescent and control cells. The level of most proteins identified in EVs changed only moderately or slightly (FC between 0.5 and 2) (Fig. 2a). Contrary to EVs, changes in the level of soluble proteins (sSASP) were more remarkable (proteins with FC > 2 or FC < 0.5). Moreover, we noticed that majority of proteins were decreased in sSASP comparing to control. This decrease was more pronounced among proteins secreted by cells undergoing replicative senescence (Fig. S2).

The proteomic composition of secretomes differed between SIPS and RS cells. We plotted differentially abundant proteins across the EVs samples using the first two PCA dimensions. The mass spectrometry (MS) samples separated into distinct categories — control, SIPS, and RS, although there was one outlier (sample H2) (Fig. S3). Among significantly increased proteins, 30% were common to EVs secreted by both types of senescent cells, while almost two times more proteins were upregulated only in EVs secreted by SIPS cells. More than 40% of proteins, the level of which decreased in senEVs, were common to EVs secreted by cells undergoing either type of senescence (SIPS and RS). However, two times more of decreased proteins were found exclusively in SIPS-EVs (Fig. 2b). Similar number of significantly upregulated and downregulated proteins were identified in sSASP of SIPS and RS, and half of those proteins were common to both types of senescence (Fig. S2). For both, EVs and sSASP proteomes, there were very few proteins common for SIPS and RS cells that change inversely (Fig. S4).

To get insight into the detailed protein composition of EVs and sSASP, we defined groups of proteins called “TOP proteins,” that represent components common for both types or unique for each type of senescence. We selected those proteins that were identified only in EVs or sSASP and the level of which increased at least 2 times comparing to control. We also included those that changed less (fold change > 1.3) but with the highest significance (*p* < 0.001) (Table S1–S6).

**Fig. 3:**
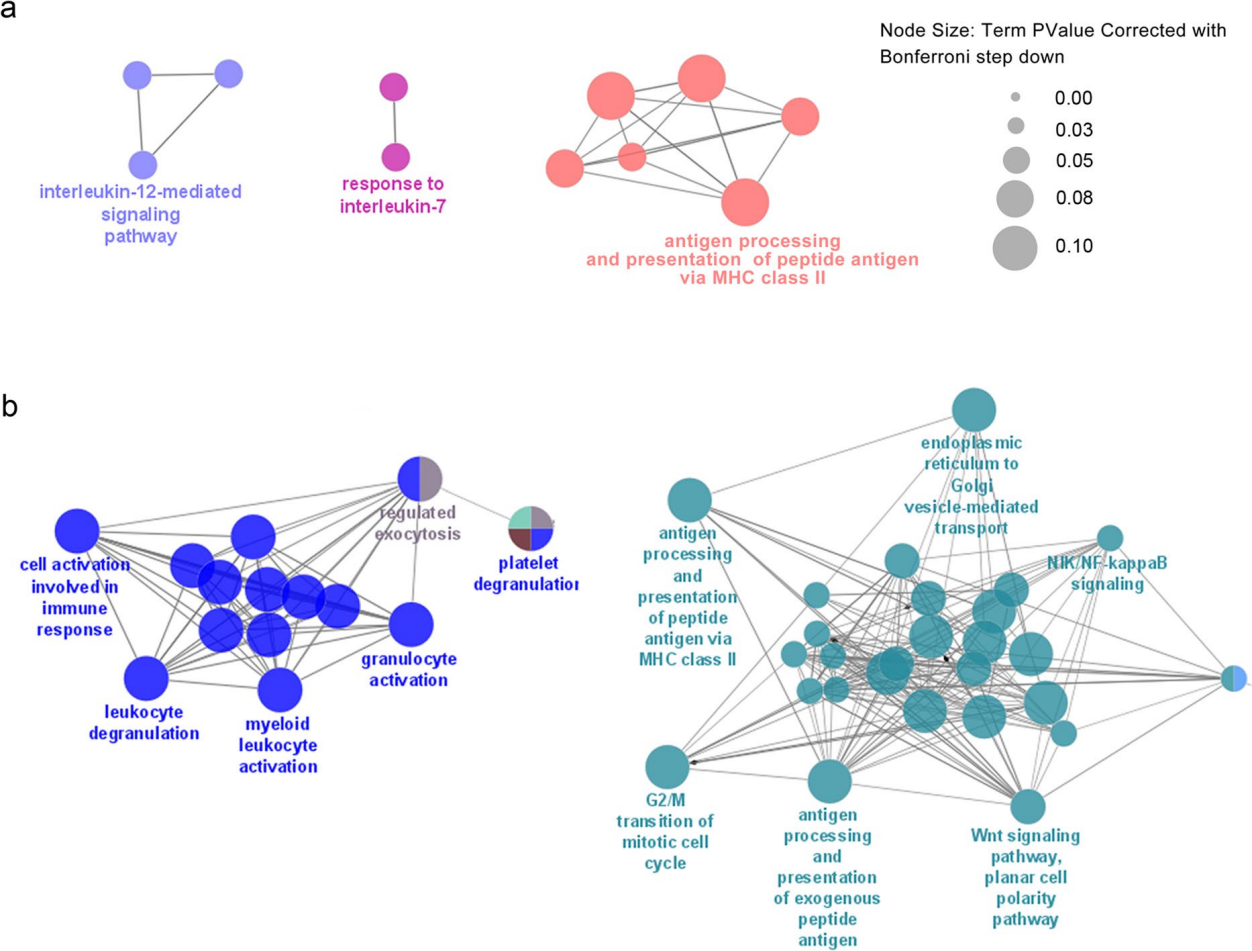
ClueGO pathway enrichment and network analysis of proteins, the level of which significantly increased in EVs secreted by VSMCs undergoing replicative (**a**) or stress induced premature senescence (**b**) and involved in immune system regulation

Reactome bioinformatics analysis of more than 200 proteins that were significantly increased (FC > 1.3) in senEVs revealed marked differences between EVs secreted by VSMCs undergoing SIPS and RS (Fig. 2c). Pathways that were the most highly represented in SIPS EVs were associated with platelet activation, signaling, and aggregation but also collagen formation and MHC class II antigen presentation, while in EVs from RS cells, they were associated with protein metabolism, scavenging by class A receptor and aggrephagy. Interestingly, proteins that were decreased in senEVs were mostly common for both types of senescence reactome pathways. Moreover, ribosomal proteins were the most numerous group of downregulated protein in senEVs (Fig. S5). Among proteins increased in EVs secreted by senescent cells, we distinguished functionally organized networks of proteins involved in immune cell function regulation (Fig. 3). The proteins assigned to ClueGO Gene Ontology Biological Process included in the networks are listed in Table S7 and S8. Based on g:Profiler tool, we distinguished proteins assigned to GO Biological Processes: Immune System Process, which increased in senEVs (Table 1).

**Table 1:** List of significantly upregulated proteins, identified in EVs secreted by senescent VSMCs, that were annotated to Immune System Process (GO:0,002,376) category; proteins that are upregulated at > 1.3- fold level are marked in bold; SIPS – stress induced premature senescence, RS – replicative senescence

Immune System Process		
SIPS and RS	only SIPS	only RS
**SERPINC1, KRT-16, HTRA1, THBS1, GAS6, FGB, DYNC1LI2, MME, PYGL, VIM, SERPINE1, PRDX2, PGM1, GSN, GLB1, CTSB, KIF5B, DPP4, GPX1**, DYNC1H1, LRP1, ACTR1A, CLTC, NECTIN2, RAB6A, ITGB3, DCTN1, TNFRSF10B, FLNA, CKAP4	**WNT5A, A2M, DNM2, CLU, C5, HBB, THBS4, PRKG1, CAPN1, PTX3, PSMB8, AIMP1, PXDN, ACTN1, RRAS, COL1A1, ZYX, DSC1, PFKL, GREM1, KLC1**, FTL, ALDOC, COL1A2, APOA4, AP2B1, C1R, SEC31L1, FTH1, C1S, TUBB, DCTN2, PKM, XRCC6, DYNC1LI1, C3, OXSR1, ANPEP, AHCY, SSC5D, MTHFD1, PSMC2, PSMD1, PSMD12, EMILIN1, CCT2, PSMC5, VAT1	**ARG1, PRSS3, TXNDC5, CAT, HRNR, CALR, PDIA3, CANX, CTSD, DSG1, CTSA, HEXB, PRDX1, PARK7, PRNP, PSMD4, APOB, PBEF1, SEC22B, PRDX3, VCP**, HSPA9, PGM3, FLNB, VCL, APEH

bold:FC>1,3

Recently, Basisty and coworkers [17] have published the results of proteomic analysis of EVs and sSASP secreted by fibroblasts and epithelial cells induced to senesce by different agents. Thus, we took the advantage of broadening our study by comparing results of proteomic analysis of the VSMC secretome with the secretome of senescent fibroblasts and epithelial cells induced to senesce by irradiation (IR). We used the same isolation procedure as Basisty et al. [17].

**Fig. 4:**
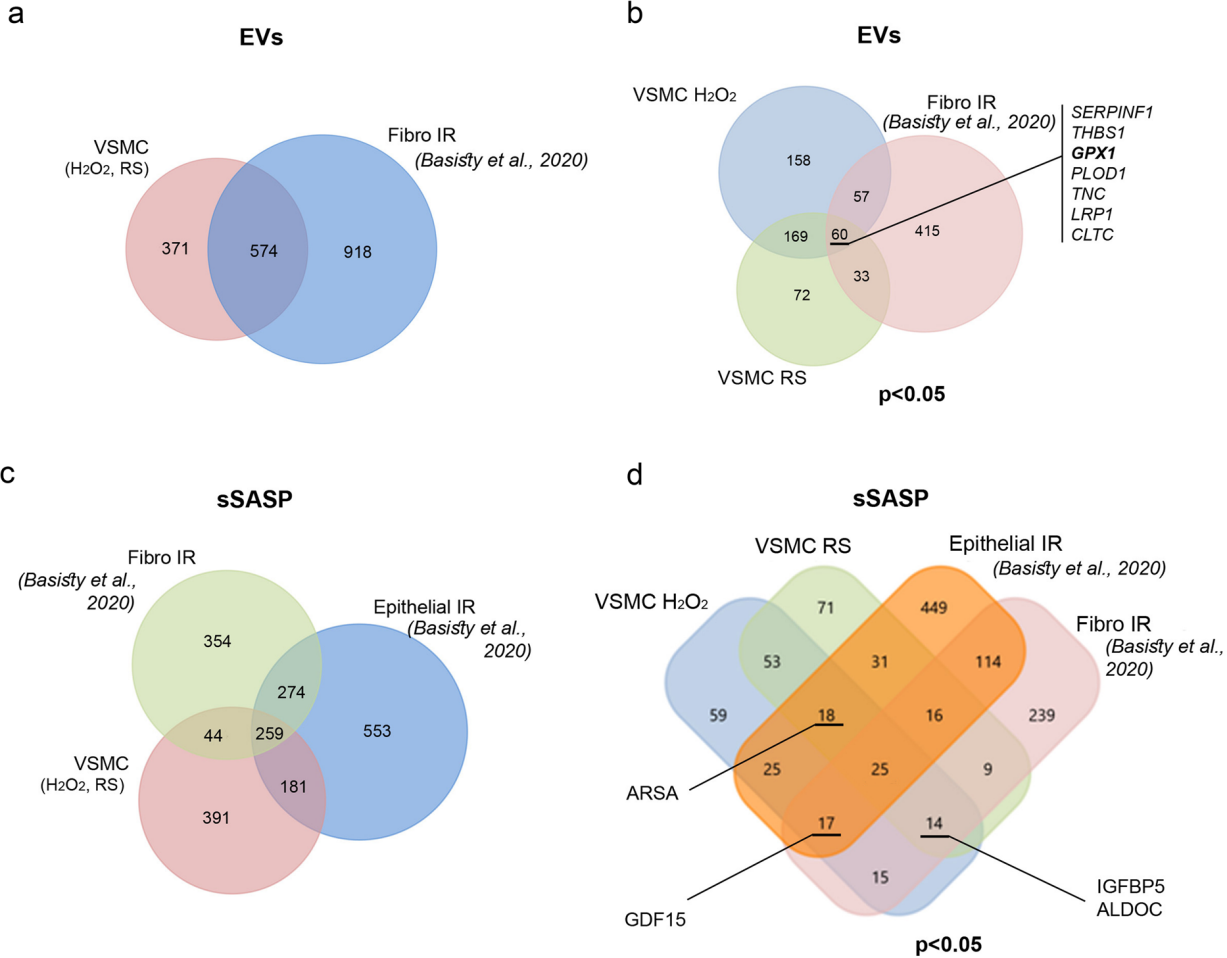
Comparison of secretome derived from senescent VSMCs with secretome of senescent fibroblast and epithelial cells (SASP atlas). **a** Venn diagram of all proteins identified in EVs secreted by senescent (RS and H_2_O_2_-induced) VSMCs and fibroblasts induced to senesce by IR (Basisty et al., 2020). **b** Venn diagram presenting proteins identified in EVs, the level of which was significantly changed (*p* < 0.05) comparing to control (young) cells. Serpin Family F Member 1 (SERPINF1), Thrombospondin 1 (THBS1), Glutathione Peroxidase 1 (GPX1), DNA Polymerase Delta 1 (POLD1), Tenascin C (TNC), LDL Receptor Related Protein 1 (LRP1), and Clathrin Heavy Chain (CLTC) are proteins that are upregulated under all conditions. **c** Venn diagram of all proteins identified in sSASP secreted by senescent (RS and H_2_O_2_-induced) VSMCs, fibroblasts induced to senesce by IR, and epithelial cells induced to senesce by IR; **d** Venn diagram presenting proteins identified in sSASP, the level of which is significantly changed (*p* < 0.05) comparing to control (young) cells. Common proteins that are upregulated under indicated conditions are listed, i.e., insulin-like growth factor-binding protein 5 (IGFB5) and fructose-bisphosphate aldolase C (ALDOC) are upregulated in fibro IR, VSMC RS, and VSMC H_2_O_2_; growth/differentiation factor 15 (GDF15) is upregulated in fibro IR, epithelial IR, and VSMC H_2_O_2_; Arylsulfatase A (ARSA) is upregulated in epithelial IR, VSMC RS, VSMC H_2_O_2_

We found that more proteins were present in EVs secreted by fibroblasts induced to senesce by irradiation than in EVs from senescent VSMCs. About 60% of proteins present in EVs secreted by senescent VSMC were also common for senescent fibroblasts (Fig. 4a). Among 574 proteins that changed significantly in senescent cells versus control, only 150 were present in EVs from VSMCs and fibroblasts. Moreover, the proteomes of EVs secreted by VSMCs undergoing SIPS and RS were more similar to each other than the proteomes of EVs secreted by VSMCs and fibroblasts that underwent premature senescence. Of note, only 7 proteins — Serpin Family F Member 1 (SERPINF1), Thrombospondin 1 (THBS1), Glutathione Peroxidase 1 (GPX1), DNA Polymerase Delta 1 (POLD1), Tenascin C (TNC), LDL Receptor Related Protein 1 (LRP1), and Clathrin Heavy Chain (CLTC) — were upregulated in all three models of senescence (Fig. 4b). Similar comparative analysis was performed for soluble proteins (sSASP) secreted by VSMCs undergoing H_2_O_2_-induced senescence and replicative senescence and by epithelial cells and fibroblasts induced to senesce by IR. We demonstrated that about one-third of all identified proteins present in VSMC sSASP were common to sSASP of fibroblasts and epithelial cells; however, the proteome of VSMCs were considerably more similar to the proteome of epithelial cells (Fig. 4c). Interestingly, less than 10% of proteins common to all three types of senescent cells changed significantly, furthermore only few of them were upregulated and listed on the Venn diagram (Fig. 4d). Detailed information about proteins common to EVs and sSASP secretomes of VSMCs, fibroblasts, and epithelial cells is presented in Table S9.

### EVs derived from senescent cells decrease activation of T lymphocytes

**Fig. 5:**
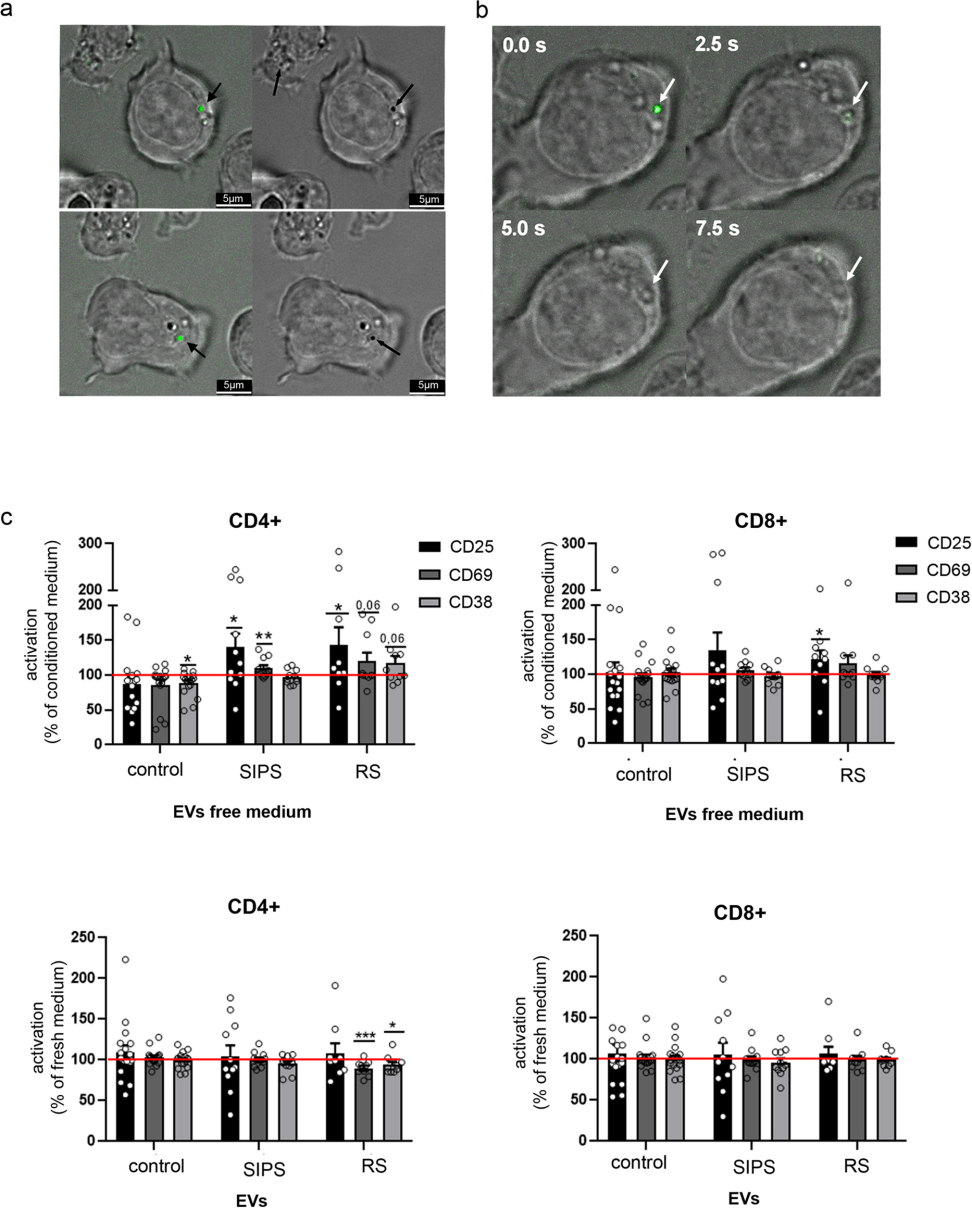
The influence of VSMCs-EVs on T cell activation. **a** Visualization of the uptake of VSMC-EVs by CD3 + cells. T cells were cocultured with CFSE stained EVs for 4 h in 37 °C and analyzed in bright-field merged with fluorescence microscopy. **b** Time laps analysis of CFSE stained EVs incorporated by T cells; **c** Expression of CD25, CD69, and CD38 in CD4 + and CD8 + subsets of T cells activated in the presence of EV-free medium or medium supplemented with EVs collected from control and senescent VSMCs. The number of cells expressing CD25, CD69, or CD38 was measured after 24 h of activation and normalized to the number of activated T cells in full conditioned medium (medium containing EVs). T cells isolated from at least 9 donors were analyzed; for statistical analysis *t*-test was used

Our proteomic analysis identified proteins upregulated in senEVs that are involved in the regulation of immune cell function; therefore, we analyzed the influence of senEVs on T cells and monocytes. To visualize the interaction of VSMC-derived EVs with lymphocytes, T cells were isolated from buffy coats and the purity of isolated CD3 + cell fraction was confirmed by flow cytometry (Fig. S6). EVs were stained with CFSE and co-cultured with T cells for 4 h at 37 °C; thereafter, live imaging was performed. We observed CFSE positive vesicles on the surface of T cells (black arrowhead) (Fig. 5a). Phase contrast analysis of the cells revealed altered structure of cytoplasmic membrane at the site of EV-cell contact, suggesting that there was a direct interaction between them (black arrows) (Fig. 5a). Moreover, we were able to visualize time-dependent internalization of EVs into T cells (Fig. 5b).

Thereafter, we analyzed the influence of EVs on T cell activation. T cells were activated in (i) conditioned medium (medium obtained after 24 h culture of control or senescent VSMCs); (ii) medium containing only soluble factors, devoid of EVs (EV-free medium); or (iii) fresh medium supplemented with EVs secreted by control or senescent cells. The percentage of cells expressing a given activation marker was measured after 24 h of culture using flow cytometry (Figs. S7, S8) and relativized to the percentage of CD25 + , CD69 + , or CD38 + cells in conditioned medium (CM). We observed that activation of T cells in EV-free medium led to increased expression of CD25 in CD4 + and CD8 + cells as well as CD69 in CD4 cells. Importantly, EV-free medium from control cells slightly decreased the number of lymphocytes that expressed activation markers. Enrichment of fresh medium with EVs secreted by RS VSMCs caused decrease in the number of CD4 + CD69 + and CD4 + CD38 + cells, while no significant influence of SIPS EVs and control EVs was observed (Fig. 5c).

### EVs derived from senescent VSMCs increase secretion of proinflammatory cytokines produced by T cells and monocytes

**Fig. 6:**
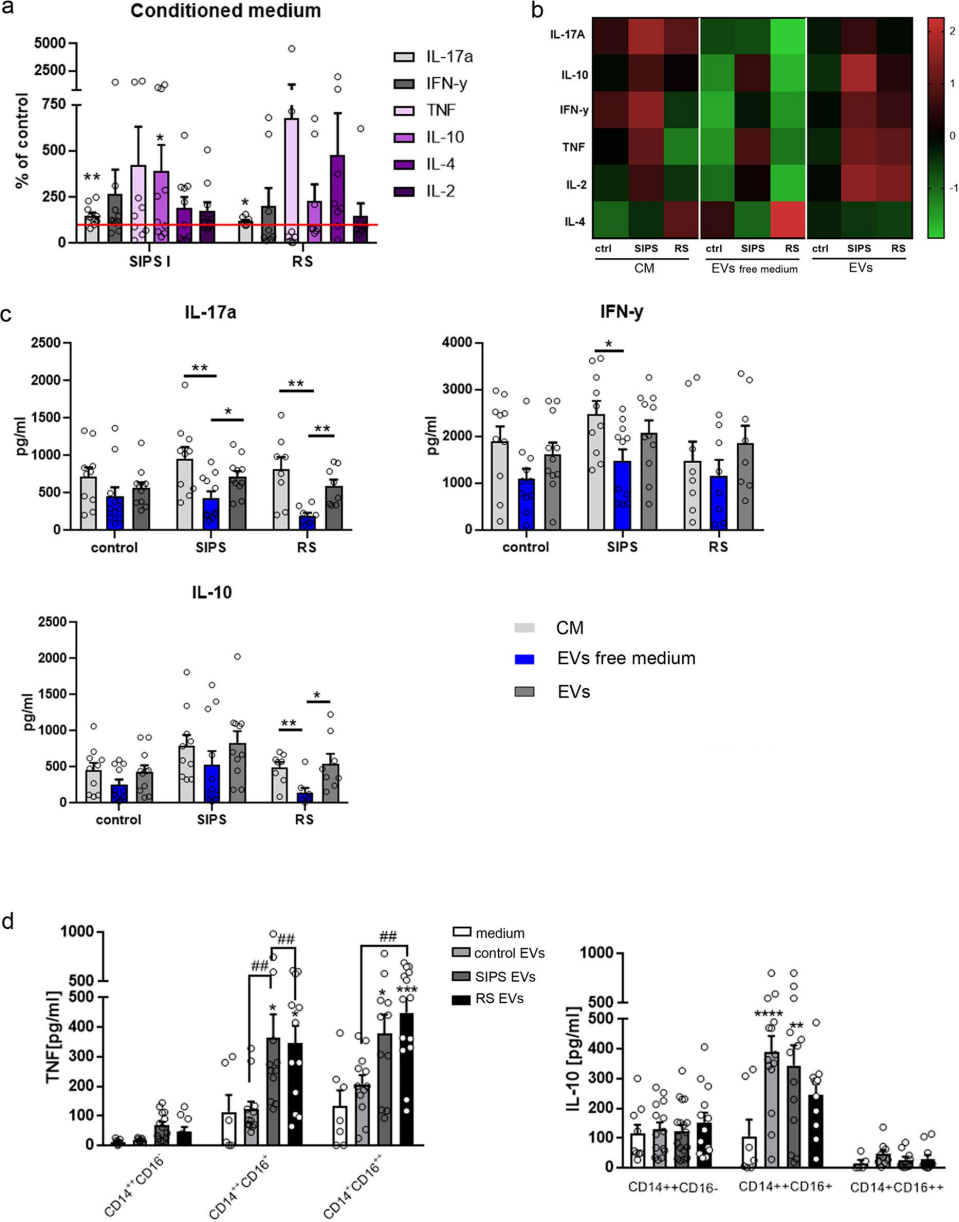
Influence of EVs secreted by VSMCs on cytokine produced by T cells and monocytes. **a** Relative amount of IL-17A, IL-10, INFγ, TNF, IL-2, and IL-4 secreted by T cells activated in the presence of senescent cells (SIPS and RS) conditioned medium (CM). The amount of each cytokine was relativized to the amount of this cytokine produced by T cells activated in control VSMC conditioned medium. Cytokines were analyzed after 72 h of culture of T cells using Cytometric Bead Array (CBA). **b** The heatmap presenting relationship between the levels of cytokines secreted by T cells upon culture in VSMC medium (CM, EV-free, and EV-enriched medium form control and senescent VSMCs). CM, EVs free medium, and EVs were collected from the same number of control, SIPS, and RS VSMCs. Original data points were standardized into the z-score. **c** The amount of selected cytokines secreted by T cells upon different culture conditions. T cells isolated from at least 8 donors were subjected into cytokine secretion measurement. Samples were analyzed using one-way ANOVA and post hoc Bonferroni test. **d** The CD14 +  + CD16-, CD14 +  + CD16 + , and CD14 + CD16 +  + monocyte subpopulations were isolated from the whole population of monocytes by cell sorting and cultured overnight in the presence of EVs derived from control and senescent VSMCs added to the culture medium. The amount of TNF and IL-10 was estimated after 18 h of culture in the medium. Monocytes isolated from at least 8 donors were subjected into cytokine secretion measurement. Samples were analyzed using one-way ANOVA and post hoc Bonferroni test, ##*p* < 0.01, *p < 0.05, **p < 0.01, ***p < 0.001, ****p < 0.0001 compared to medium

We examined the effect of EVs on secretion of cytokines by CD3 + lymphocytes. To this end, we first checked the level of cytokines (IL17A, IL-10, INFγ, TNFα, IL-4, IL-2) using Cytometric Bead Array (CBA) in the CM collected from VSMC culture and found they were undetectable either in control cells or senescent cells CM (data not shown). Thereafter, T cells were activated in the presence of CM from control and senescent VSMCs, EV-free medium, or fresh medium supplemented with EVs. After 72 h, medium from the T cell culture was collected and the amount of secreted cytokines was measured using CBA. Both SIPS and RS conditioned medium led to increased secretion of cytokines when compared to T cells cultured in the control cells conditioned medium (Fig. 6a). The most consistent results were obtained for IL-17A, the level of which increased to 150% (SIPS CM) and 120% (RS CM) of that in control cells CM. Apart from that, T cells cultured in SIPS CM secreted significantly more IL-10. To estimate the role of EVs in the induction of cytokine secretion, we compared changes in the secretion of cytokines when T cells were cultured in conditioned medium devoid of EVs or in the fresh medium supplemented with EVs derived from control, SIPS, or RS cells. Our studies revealed that removal of EVs from conditioned medium decreased the secretion level of most tested cytokines (IL17A, IL-10, INFγ, TNFα, IL-2), while culturing T cells in the presence of EVs led to upregulation of cytokine secretion (Fig. 6b). Importantly, senEVs (both SIPS and RS) have a clearly higher impact than control EVs (Fig. 6b). Of note, changes observed in the level of cytokines secreted by T cells cultured in EV-free medium, or EVs from SIPS and RS VSMCs, were statistically significant for IL-17A (Fig. 6c). When T cells were cultured in EVs free medium from senescent VSMCs, the amount of IL-17 secreted by T cells was decreased by half (56%) for SIPS and by 75% for RS comparing to conditioned medium (medium containing EVs and soluble factors). Re-application of senEVs into the fresh medium led to statistically significant upregulation of IL-17 secretion to the level of 75% (SIPS EVs) and 72% (RS EVs) of that measured for T cells cultured in conditioned medium. IL-10 secretion was significantly modified when T cells were cultured in the presence or absence of EVs collected form RS cells, while the level of INFγ decreased significantly when T cells were cultured in EV-free medium from SIPS cells (Fig. 6c).

We also estimated the influence of senEVs on cytokine production by monocytes. Secretion of cytokines was determined after 18 h culture of sorted monocyte subpopulations with control or senescent EVs. Control EVs did not induce secretion of TNF in any of the monocyte subpopulations, while senescent EVs significantly elevated secretion of TNF by CD14 +  + CD16 + and CD14 + CD16 +  + cells. In contrast, IL-10 was secreted mainly by an intermediate (CD14 +  + CD16 +) subpopulation of monocytes to a similar extent in response to control EVs and senEVs (Fig. 6d).

### EVs derived from VSCMs increase expression of CCR2 and CD16 by MDM

To estimate the influence of EVs derived from senescent VSMCs, MDM were differentiated in the medium supplemented with EVs derived from control and senescent VSMCs. MDM differentiated in the presence of EVs derived from senescent VSCMs (SIPS EVs, RS EVs) expressed significantly more CCR2 than MDM exposed to control EVs. Expression of CD16 was significantly elevated when MDM were cultured in the presence of SIPS EVs in comparison with control EVs. Altogether, this finding shows that senEVs influenced expression of selected differentiation markers without unequivocally promoting either the M1 or M2 phenotype (Fig. S9). We also noticed increased secretion, however without statistical significance, of TNF in MDM differentiated in the presence of SIPS EVs (not shown).

## Discussion

Secretion of bioactive factors by senescent cells has been widely recognized as one of the major contributors to the development of aging and age-related diseases. Although senescence associated secretory phenotype (SASP) is a hallmark of cellular senescence, we have just started to realize its complexity and diversity as well as its role in the modulation of the microenvironment in a given cellular context.

In 2008, Lehmann et al. demonstrated for the first time increased EV secretion by senescent prostate cancer cells. Later on, subsequent studies confirmed a similar phenomenon in other cell types, mostly fibroblasts [17, 18] and HUVEC [19]. We are the first to show that VSMCs, which underwent SIPS or RS, released higher number of EVs than non-senescent cells. Moreover, based on the expression of protein markers, we were able to prove that a fraction of secreted EVs is enriched in exosomes. Similar observation has been described recently by Mensa et al. (2020) [20], who showed that senescent endothelial cells secret more exosomes than non-senescent cells. Altogether, those results prove that increased secretion of exosomes is a universal feature of SASP.

Here, we demonstrate the results of an unbiased proteomic analysis of senescent VSMC secretome. Since EVs have already been shown to contribute to SASP, we separated vesicular and soluble fraction of the secretome to perform mass spec analysis. Our studies revealed significant differences in proteomic composition of SASP depending on the type of senescence (H_2_O_2_-induced versus replicative senescence). Interestingly, we have demonstrated that at least half or more proteins identified in SASP are downregulated comparing to the secretome of control cells. This observation proves that increased level of secretion by senescent cells is not always accompanied by an increased level of proteins that participate in SASP. This phenomenon may reflect a drop in the global translation level, which was described for mouse fibroblasts undergoing replicative senescence [21]. On the contrary, studies performed on human fibroblasts induced to senescence by Ras overexpression or etoposide treatment demonstrated that majority of the identified proteins were upregulated comparing to EVs secreted by control cells [22]. Moreover, we found out that majority of the most significantly downregulated proteins in SASP were ribosomal proteins and those assigned to translation and ribosomes’ function reactome pathways. Since a decrease in ribosomal biogenesis [23] and expression of ribosomal proteins [24] in senescent cells has already been recognized, the apparent decline of their content in SASP confirms reorganization of the translation apparatus in senescent VSMCs. Interestingly ribosomal proteins have been recognized by others as an EVs cargo [25]. For instance EVs secreted by LPS-mediated microglia were enriched in ribosomal proteins [26]. Different ribosomal proteins were also identified in the exosomes of ischemia challenged epicardial adipose tissue–derived stem cells [27]. The biological meaning of those proteins present in EVs is still unrevealed. However, there are a number of papers confirming the extraribosomal function of proteins building ribosomes. Ribosomal protein L13a was shown to negatively regulate transcription of pro-inflammatory cytokine encoding mRNA [28, 29] leading to attenuation of inflammation. Another ribosomal protein — RPS19 has been shown to exert anti-inflammatory function by blocking a pro-inflammatory macrophage cytokine — MIF, which in consequence, modulated important immune cell functions studied in a mouse model of severe inflammatory renal injury [30]. Thus we consider that changes in the ribosomal protein content of senEVs, that were recognized by us, may influence immune cell function as well as exert a pro-inflammatory response. Moreover, we noticed that the protein composition of EVs secreted by VSMCs undergoing RS is more similar to that of control cells than to that of SIPS cells. This observation indicates that the rapid and timely coordinated process of SIPS is reflected in more pronounced changes in secretome composition.

We compared the composition of VSMC secretome with SASP atlas data created by Basisty [17] for fibroblasts and epithelial cells and demonstrated marked differences in SASP composition between different cell types. Nevertheless, we identified seven proteins that were commonly increased in EVs secreted by senescent fibroblasts and VSMCs. Among them were SERPIN F1 and thrombospondin 1 (THBS1). Importantly, serine protease inhibitors — Serpins as well as thrombospondin 1 — have recently been shown to belong to the SASP factors that facilitate increased blood clotting in mice treated with doxorubicin to induce cell senescence [31]. Accordingly, elimination of senescent cells in dox-treated p16-3MR mice led to reversion of the hemostatic effect. Moreover, proteomic analysis of secretom of senescent fibroblasts confirmed the presence of proteins involved in blood coagulation in senEVs [22]. SERPIN F1 represents a member of the Serpin family that does not possess serine protease inhibitory function. Instead, it was demonstrated that SERPIN F1 has anti-angiogenic, pro-apoptotic and cell cycle inhibitory activity involving several distinct pathways [32]. The role of thrombospondin in aging might also go beyond blood clotting regulation, since its increased expression has been correlated with several age-related diseases such as myocardial infraction, heart failure, pulmonary arterial hypertension, and glaucoma [33]. Moreover, THBS1 may activate TGFβ and both were shown to be secreted by senescent peritoneal mesothelial cells leading to TGFβ-dependent spreading of the senescence phenotype [34]. Beside TGFβ activation, THSB1 might upregulate expression and activity of IL-1β, as it was demonstrated in LPS-stimulated monocyte-derived macrophages [35]. On the other hand IL-1β induce p16 expression due to miR-24 repression leading to cell senescence [36]. THBS1 can also induce senescence by upregulating Nox1-produced ROS, triggering DNA damage and DDR activation in human endothelial cells [37]. Thus, THBS1 might represent a common SASP factor that acts as a mediator of aging.

Comparison of soluble factors secreted by senescent VSMCs with the secretome of fibroblasts and epithelial cells analyzed by Basisty et al. [17] allowed us to reveal GDF15 as the only protein commonly increased in the secretome of those different cell types induced to senesce by H_2_O_2_ or IR. Importantly, GDF15 was also indicated as one out of four proteins that were identified as common SASP components for fibroblasts to senesce by diverse factors [17] and a protein marker of human aging detected in the plasma [38]. GDF15 expression is highly upregulated in the atherosclerotic vessel wall and lack of GDF15 resulted in a significant long-term reduction of atherosclerotic lesion formation [39]. Altogether, our proteomic analysis of the VSMC secretome, complemented by the SASP atlas data [17], allowed us to point out the universal components of SASP present in EVs or secreted as soluble proteins, that might be tested as markers of senescence and aging. On the other hand, we assigned a group of proteins (“top” proteins) most remarkably and significantly altered in EVs and sSASP of senescent VSMCs, which might represent more cell type specific components of SASP. Still, few among those proteins (SERPINC1, SERPINA7, Complement factor I CFI, Prothrombin F2) represent factors involved in hemostasis regulation. Accordingly, reactome pathway analysis confirmed marked enrichment of pathways involved in platelet activation and coagulation in senEVs derived proteins. Importantly, in the course of atherosclerosis development, increased thrombosis at the site of vulnerable plaques increases the risk of ischemic events. Therefore, medical recommendations of therapy for the prevention of recurrent atherothrombosis include anti-thrombotic drugs [40]. Since senescent cells were shown to accumulate in the atherosclerotic plaque we might speculate, on the basis of the analysis of VSMC SASP composition, that another important and deleterious role of senescent cells would be local enhancement of thrombosis.

Atherosclerosis is an inflammatory disease characterized by intense immunological activity. Immune cells infiltrate from the blood into the plaque and contribute to plaque development, promoting local inflammation. Numerous studies have indicated T cells as critical drivers and modifiers of the pathogenesis of atherosclerosis. Importantly, EVs released by leukocytes, platelets, smooth muscle cells (SMCs), and endothelial cells have been shown to promote vascular inflammation and atherosclerosis [41]. There is also a growing body of evidence that EVs secreted by senescent cells can modulate the functioning of other cells. Recently, an elegant study performed by Borghesan et al. [22] have demonstrated that senEVs secreted by senescent human dermal fibroblasts are responsible for induction of paracrine senescence in other cells. This phenomenon was attributed to Interferon-induced transmembrane protein 3 (IFITM3), the level of which is increased in senEVs. Thus, we analyzed the impact of EVs derived from senescent VSMCs on T cell activation. Recent studies revealed that the CD4 + and CD8 + T cell subsets in the plaque are more activated than their blood counterparts, presenting a heterogeneous spectrum of activation [42]. Significant differences were noted for CD8 + cells expressing CD25 and CD38 markers, while in the case of CD4 + cells only CD38 expression was discriminative between plaque and blood lymphocytes [43]. Unexpectedly, our studies revealed that depletion of EVs from senescent cell conditioned medium increased the level of activation markers suggesting that senEVs restrict T cell activation.

We estimated the influence of control and senEVs on lymphocyte polarization, based on their cytokine production. Numerous studies performed on mouse experimental models, as well as ex vivo samples from patients, revealed the presence in the plaque of different subpopulations of T cells that had either proatherogenic or antiatherogenic potential. In this context the influence of factors secreted by senescent VSMCs that participate in plaque development remains unknown. We demonstrated that factors released by senescent VSMCs and present in conditioned medium increase the level of IL-17 secreted by CD3 + cells. Furthermore, analysis performed with conditioned medium deprived of EVs as well as fresh medium supplemented with EVs proved that EVs secreted by senescent VSMCs upregulate the production of IL-17 by T cells. Interestingly, higher level of IL-17A and IFN-γ was also demonstrated in the culture of human T cells derived from the plaque comparing to T cells isolated from non-diseased vessels after polyclonal stimulation [44]. The proatherogenic role of IL-17 in mouse models has been revealed; however, there are also opposite results proving antiatherogenic activity of IL-17 [45]. Studies performed in humans are also ambiguous, indicating either detrimental [46, 47] or beneficial [48] role of this cytokine in atherosclerosis. Of note, EVs derived from senescent VSMCs were shown to induce also the secretion of INF-γ (SIPS) and IL-10 (RS). Those two cytokines exert opposite effects. INF-γ is a proinflammatory cytokine. Accordingly, a proatherogenic function is ascribed to the IFN-γ-producing T helper 1 (Th1) subset, whereas the interleukin IL-10-producing regulatory T cells (Tregs) are atheroprotective [49]. Although in-depth analysis is needed to unequivocally prove that EVs secreted by senescent VSMCs promote differentiation into a particular T cell subtype, we demonstrated that senEVs might influence production of cytokines by T cells and, in this way, actively modulate the cytokine milieu of the plaque. The final outcome of this intracellular communication will certainly depend on the complex interaction between plaque building cells.

Although there are a number of proteins identified in senEVs that might potentially regulate immune cell function, alone or in combination, one of the potential candidates responsible for the observed effect is thrombospondin-1 (THSB1). Its level was increased in EVs secreted by SIPS and RS VSMCs. Thrombospondin can play pro-inflammatory but also an anti-inflammatory role in several diseases, depending on its interaction with multiple receptors as well as the presence of specific matricellular proteins [50]. Interestingly, THSB1 was shown to act as an inhibitor of TCR-mediated T cell activation. Treatment of CD3-stimulated human T cells with THSB1 resulted in downregulation of CD69 expression and IL-2 level [51]. However, a contradictory effect of THSB1 on T cell activation has also been reported [52]. Furthermore, it was demonstrated that exosomes secreted by LPS-treated human thymic mesenchymal stromal cells (tMSCs) contain increased level of THSB1 comparing to untreated tMSCs. Culturing of CD4 + lymphocytes with LPS-tMSC-derived exosomes caused inhibition of T cell proliferation and promoted differentiation of CD4 + into Th1 and Th17 cells [53].

Monocytes and macrophages are the most common immune cells in an expanding atherosclerotic plaque. They play a role in cholesterol accumulation, lesion matrix remodeling, cytokine production, and clearance of dead cell debris. Monocytes from the blood infiltrate into the plaque and might differentiate into inflammatory or anti-inflammatory macrophages depending on the cytokine availability and some local metabolic cues. Still little is known about tissue-specific adaptation of monocytes within the atherosclerotic plaque. We demonstrated that senescent EVs significantly increase expression of CCR2—receptor for monocyte chemoattractant protein-1 (MCP-1), on the surface of MDMs. Interestingly, expression of CCR2 has been shown to play a critical role in atherosclerosis development. It was demonstrated that there is a significant decrease of lesion formation in ApoE-/- mice that lack the expression of CCR2 (ApoE-/- CCR2-/-). CCR2 was important in recruitment of monocytes/macrophages into the vessel wall [54]. Moreover, EVs derived from SIPS VSMCs also induce the expression of CD16 on MDMs. Thus, results of our studies suggest that EVs secreted by senescent VSMCs favor differentiation of monocytes with mixed M1/M2 polarization with proinflammatory characteristics.

We also analyzed the influence of EVs on cytokine production in different subtypes of monocytes and demonstrated that senEVs significantly enhanced secretion of TNF-α in intermediate (CD14 +  + CD16 +) and non-classical (CD14 + CD16 + +) monocytes. The TNF-α cytokine was previously shown to a play critical role in atherosclerosis development [55] while TNF-α deficiency has been associated with decreased plaque inflammation [56]. Among different monocyte subtypes, CD16 + (both intermediate and non-classical) are the main producers of TNF-α [57]. The expansion of the CD16 + monocytes has been documented in many different diseases, mostly in infection or inflammatory conditions [58–62]. Expansion of intermediate and non-classical monocytes during aging has been also reported [63, 64]. Moreover CD16 + monocytes from aged individuals were shown to produce higher level of TNFα upon LPS stimulation compared to young individuals [65]. There is also a growing body of evidence demonstrating a significant role of CD16 + monocytes (both intermediate and non-classical) in the development of atherosclerosis [66]. CD14 + CD16 + monocyte increased number was associated with diagnosis of coronary atherosclerosis. Also, patients with the highest levels of CD14 + CD16 + monocytes had increased serum concentrations of TNF-α [67]. Other studies revealed, that there is a relationship between enhanced levels of CD16 + monocytes and the presence of vulnerable atherosclerotic plaques, either in patients with stable or unstable angina pectoris [68]. Studies performed on a group of over 900 subjects from the general population at cardiovascular risk revealed that, after full adjustment for cardiovascular risk factors, only the CD14 +  + CD16 + intermediate monocyte subtype remained an independent predictor of adverse cardiovascular outcomes [69]. On the other hand, there are also studies showing contradictory results [70]; thus, the role of particular monocyte subsets needs further investigation. In sum, our results support the inflammatory potential of senEVs secreted by VSMCs by way of influencing monocyte activity.

Altogether, we have demonstrated for the first time that EVs secreted by senescent VSMCs can modify functioning of different types of immune cells that play a prominent role in atherosclerosis development and progression. Our studies broaden and enrich the observation of Gardner et al. [12], who revealed that factors secreted by senescent cells induced chemotaxis of monocyte-like cells (THP-1) in vitro. The effect was dependent on IL-1α and MCP-1 secreted by senescent VSMCs. They also demonstrated that senescent VSMCs injected into the mouse cavity led to recruitment of higher number of leukocytes and monocytes/macrophages. Moreover, immunocytochemical analysis of human carotid plaques revealed that CD68-positive cells accumulate in close proximity to senescent cells, suggesting that those cells attract monocytes/macrophages. Further studies performed in mouse that stably expressed mutated telomeric repeat-binding factor 2 protein (TRF2^T188A^) in VSMCs showed increased number of senescent cells in the vessel wall after injury. In consequence, SASP factors secreted by senescent VSMCs caused increased recruitment of immune cells [71]. Results of our study indicate that SASP components secreted by senescent VSMCs may facilitate inflammation also by stimulating proinflammatory cytokine production by T cells and monocytes as well as by triggering a proinflammatory phenotype of macrophages. Altogether, these results strengthen the role of senescent cells as an important contributor to inflammation in atherosclerosis.

## Supplementary Information

Below is the link to the electronic supplementary material.

Supplementary file1 (PDF 1555 KB)

Supplementary file2 (PDF 237 KB)

Supplementary file3 (PDF 223 KB)

Supplementary file4 (PDF 971 KB)

## Data Availability

The mass spectrometry proteomics data are available via ProteomeXchange with identifier PXD030955. Reviewer account details: Username: reviewer_pxd030955@ebi.ac.uk; Password: 6StTO0yk.

## References

[1] Libby P (2012). Inflammation in atherosclerosis. Arterioscler Thromb Vasc Biol.

[2] Matthews C, Gorenne I, Scott S, Figg N, Kirkpatrick P, Ritchie A, Goddard M, Bennett M (2006). Vascular smooth muscle cells undergo telomere-based senescence in human atherosclerosis: effects of telomerase and oxidative stress. Circ Res.

[3] Costopoulos C, Liew TV, Bennett M (2008). Ageing and atherosclerosis: mechanisms and therapeutic options. Biochem Pharmacol.

[4] Gorenne I, Kavurma M, Scott S, Bennett M (2006). Vascular smooth muscle cell senescence in atherosclerosis. Cardiovasc Res.

[5] Childs BG, Baker DJ, Wijshake T, Conover CA, Campisi J, van Deursen JM (2016). Senescent intimal foam cells are deleterious at all stages of atherosclerosis. Science.

[6] Coppe JP, Desprez PY, Krtolica A, Campisi J (2010). The senescence-associated secretory phenotype: the dark side of tumor suppression. Annu Rev Pathol.

[7] Wallis R, Mizen H, Bishop CL (2020). The bright and dark side of extracellular vesicles in the senescence-associated secretory phenotype. Mech Ageing Dev.

[8] Raposo G, Stoorvogel W (2013). Extracellular vesicles: exosomes, microvesicles, and friends. J Cell Biol.

[9] Konkoth A, Saraswat R, Dubrou C, Sabatier F, Leroyer AS, Lacroix R, Duchez AC, Dignat-George F (2021). Multifaceted role of extracellular vesicles in atherosclerosis. Atherosclerosis.

[10] Rautou PE, Vion AC, Amabile N, Chironi G, Simon A, Tedgui A, Boulanger CM (2011). Microparticles, vascular function, and atherothrombosis. Circ Res.

[11] Yin M, Loyer X, Boulanger CM (2015). Extracellular vesicles as new pharmacological targets to treat atherosclerosis. Eur J Pharmacol.

[12] Gardner SE, Humphry M, Bennett MR, Clarke MC (2015). Senescent vascular smooth nuscle cells drive inflammation through an interleukin-1alpha-dependent senescence-associated secretory phenotype. Arterioscler Thromb Vasc Biol.

[13] Thery C, Amigorena S, Raposo G, Clayton A (2006). Isolation and characterization of exosomes from cell culture supernatants and biological fluids. Curr Protoc Cell Biol.

[14] Baj-Krzyworzeka M, Mytar B, Szatanek R, Surmiak M, Weglarczyk K, Baran J, Siedlar M (2016). Colorectal cancer-derived microvesicles modulate differentiation of human monocytes to macrophages. J Transl Med.

[15] Wisniewski JR, Ostasiewicz P, Mann M (2011). High recovery FASP applied to the proteomic analysis of microdissected formalin fixed paraffin embedded cancer tissues retrieves known colon cancer markers. J Proteome Res.

[16] Przybylska D, Janiszewska D, Gozdzik A, Bielak-Zmijewska A, Sunderland P, Sikora E, Mosieniak G. NOX4 downregulation leads to senescence of human vascular smooth muscle cells. Oncotarget. 2016; 10.18632/oncotarget.12079. 10.18632/oncotarget.12079 PMC5341811 27655718

[17] Basisty N, Kale A, Jeon OH, Kuehnemann C, Payne T, Rao C, Holtz A, Shah S, Sharma V, Ferrucci L, Campisi J, Schilling B (2020). A proteomic atlas of senescence-associated secretomes for aging biomarker development. PLoS Biol.

[18] Takahashi A, Okada R, Nagao K, Kawamata Y, Hanyu A, Yoshimoto S, Takasugi M, Watanabe S, Kanemaki MT, Obuse C, Hara E (2017). Exosomes maintain cellular homeostasis by excreting harmful DNA from cells. Nat Commun.

[19] Riquelme JA, Takov K, Santiago-Fernandez C, Rossello X, Lavandero S, Yellon DM, Davidson SM (2020). Increased production of functional small extracellular vesicles in senescent endothelial cells. J Cell Mol Med.

[20] Mensa E, Guescini M, Giuliani A, Bacalini MG, Ramini D, Corleone G, Ferracin M, Fulgenzi G, Graciotti L, Prattichizzo F, Sorci L, Battistelli M, Monsurro V, Bonfigli AR, Cardelli M, Recchioni R, Marcheselli F, Latini S, Maggio S, Fanelli M, Amatori S, Storci G, Ceriello A, Stocchi V, De Luca M, Magnani L, Rippo MR, Procopio AD, Sala C, Budimir I, Bassi C, Negrini M, Garagnani P, Franceschi C, Sabbatinelli J, Bonafe M, Olivieri F. Small extracellular vesicles deliver miR-21 and miR-217 as pro-senescence effectors to endothelial cells. J Extracell Vesicles. 2020; 10.1080/20013078.2020.1725285. 10.1080/20013078.2020.1725285 PMC7048230 32158519

[21] Wu S, Xu S, Li R, Li K, Zhong X, Li Y, Zhou Z, Liu Y, Feng R, Zheng J, Songyang Z, Liu F (2019). mTORC1-Rps15 axis contributes to the mechanisms underlying global translation reduction during senescence of mouse embryonic gibroblasts. Front Cell Dev Biol.

[22] Borghesan M, Fafian-Labora J, Eleftheriadou O, Carpintero-Fernandez P, Paez-Ribes M, Vizcay-Barrena G, Swisa A, Kolodkin-Gal D, Ximenez-Embun P, Lowe R, Martin-Martin B, Peinado H, Munoz J, Fleck RA, Dor Y, Ben-Porath I, Vossenkamper A, Munoz-Espin D, O'Loghlen A (2019). Small extracellular vesicles are key regulators of non-cell autonomous intercellular communication in senescence via the interferon protein IFITM3. Cell Rep.

[23] Lessard F, Igelmann S, Trahan C, Huot G, Saint-Germain E, Mignacca L, Del Toro N, Lopes-Paciencia S, Le Calve B, Montero M, Deschenes-Simard X, Bury M, Moiseeva O, Rowell MC, Zorca CE, Zenklusen D, Brakier-Gingras L, Bourdeau V, Oeffinger M, Ferbeyre G (2018). Senescence-associated ribosome biogenesis defects contributes to cell cycle arrest through the Rb pathway. Nat Cell Biol.

[24] Seshadri T, Uzman JA, Oshima J, Campisi J. Identification of a transcript that is down-regulated in senescent human fibroblasts. Cloning, sequence analysis, and regulation of the human L7 ribosomal protein gene. J Biol Chem. 1993, https://www.ncbi.nlm.nih.gov/pubmed/8360149. 8360149

[25] Yokoi A, Ochiya T (2021). Exosomes and extracellular vesicles: rethinking the essential values in cancer biology. Semin Cancer Biol.

[26] Yang Y, Boza-Serrano A, Dunning CJR, Clausen BH, Lambertsen KL, Deierborg T (2018). Inflammation leads to distinct populations of extracellular vesicles from microglia. J Neuroinflammation.

[27] Thankam FG, Huynh J, Fang W, Chen Y, Agrawal DK (2022). Exosomal-ribosomal proteins-driven heterogeneity of epicardial adipose tissue derived stem cells under ischemia for cardiac regeneration. J Tissue Eng Regen Med.

[28] Vyas K, Chaudhuri S, Leaman DW, Komar AA, Musiyenko A, Barik S, Mazumder B (2009). Genome-wide polysome profiling reveals an inflammation-responsive posttranscriptional operon in gamma interferon-activated monocytes. Mol Cell Biol.

[29] Poddar D, Basu A, Baldwin WM, Kondratov RV, Barik S, Mazumder B (2013). An extraribosomal function of ribosomal protein L13a in macrophages resolves inflammation. J Immunol.

[30] Lv J, Huang XR, Klug J, Frohlich S, Lacher P, Xu A, Meinhardt A, Lan HY (2013). Ribosomal protein S19 is a novel therapeutic agent in inflammatory kidney disease. Clin Sci (Lond).

[31] Wiley CD, Liu S, Limbad C, Zawadzka AM, Beck J, Demaria M, Artwood R, Alimirah F, Lopez-Dominguez JA, Kuehnemann C, Danielson SR, Basisty N, Kasler HG, Oron TR, Desprez PY, Mooney SD, Gibson BW, Schilling B, Campisi J, Kapahi P (2019). SILAC analysis reveals increased secretion of hemostasis-related factors by senescent cells. Cell Rep.

[32] He X, Cheng R, Benyajati S, Ma JX (2015). PEDF and its roles in physiological and pathological conditions: implication in diabetic and hypoxia-induced angiogenic diseases. Clin Sci (Lond).

[33] Isenberg JS, Roberts DD (2020). Thrombospondin-1 in maladaptive aging responses: a concept whose time has come. Am J Physiol Cell Physiol.

[34] Mikula-Pietrasik J, Sosinska P, Janus J, Rubis B, Brewinska-Olchowik M, Piwocka K, Ksiazek K (2013). Bystander senescence in human peritoneal mesothelium and fibroblasts is related to thrombospondin-1-dependent activation of transforming growth factor-beta1. Int J Biochem Cell Biol.

[35] Stein EV, Miller TW, Ivins-O'Keefe K, Kaur S, Roberts DD (2016). Secreted thrombospondin-1 regulates macrophage interleukin-1beta production and activation through CD47. Sci Rep.

[36] Philipot D, Guerit D, Platano D, Chuchana P, Olivotto E, Espinoza F, Dorandeu A, Pers YM, Piette J, Borzi RM, Jorgensen C, Noel D, Brondello JM (2014). p16INK4a and its regulator miR-24 link senescence and chondrocyte terminal differentiation-associated matrix remodeling in osteoarthritis. Arthritis Res Ther.

[37] Meijles DN, Sahoo S, Al Ghouleh I, Amaral JH, Bienes-Martinez R, Knupp HE, Attaran S, Sembrat JC, Nouraie SM, Rojas MM, Novelli EM, Gladwin MT, Isenberg JS, Cifuentes-Pagano E, Pagano PJ (2017). The matricellular protein TSP1 promotes human and mouse endothelial cell senescence through CD47 and Nox1. Sci Signal.

[38] Tanaka T, Biancotto A, Moaddel R, Moore AZ, Gonzalez-Freire M, Aon MA, Candia J, Zhang P, Cheung F, Fantoni G, consortium CHI, Semba RD, Ferrucci L. Plasma proteomic signature of age in healthy humans. Aging Cell. 2018; 10.1111/acel.12799. 10.1111/acel.12799 PMC6156492 29992704

[39] Bonaterra GA, Zugel S, Thogersen J, Walter SA, Haberkorn U, Strelau J, Kinscherf R (2012). Growth differentiation factor-15 deficiency inhibits atherosclerosis progression by regulating interleukin-6-dependent inflammatory response to vascular injury. J Am Heart Assoc.

[40] Gallone G, Baldetti L, Pagnesi M, Latib A, Colombo A, Libby P, Giannini F (2018). Medical therapy for long-term prevention of atherothrombosis following an acute coronary syndrome: JACC State-of-the-Art Review. J Am Coll Cardiol.

[41] van der Vorst EPC, de Jong RJ, Donners M (2018). Message in a microbottle: modulation of vascular inflammation and atherosclerosis by extracellular vesicles. Front Cardiovasc Med.

[42] Fernandez DM, Rahman AH, Fernandez NF, Chudnovskiy A, Amir ED, Amadori L, Khan NS, Wong CK, Shamailova R, Hill CA, Wang Z, Remark R, Li JR, Pina C, Faries C, Awad AJ, Moss N, Bjorkegren JLM, Kim-Schulze S, Gnjatic S, Ma'ayan A, Mocco J, Faries P, Merad M, Giannarelli C (2019). Single-cell immune landscape of human atherosclerotic plaques. Nat Med.

[43] Grivel JC, Ivanova O, Pinegina N, Blank PS, Shpektor A, Margolis LB, Vasilieva E (2011). Activation of T lymphocytes in atherosclerotic plaques. Arterioscler Thromb Vasc Biol.

[44] Eid RE, Rao DA, Zhou J, Lo SF, Ranjbaran H, Gallo A, Sokol SI, Pfau S, Pober JS, Tellides G (2009). Interleukin-17 and interferon-gamma are produced concomitantly by human coronary artery-infiltrating T cells and act synergistically on vascular smooth muscle cells. Circulation.

[45] Akhavanpoor M, Akhavanpoor H, Gleissner CA, Wangler S, Doesch AO, Katus HA, Erbel C (2017). The two faces of interleukin-17A in atherosclerosis. Curr Drug Targets.

[46] Erbel C, Dengler TJ, Wangler S, Lasitschka F, Bea F, Wambsganss N, Hakimi M, Bockler D, Katus HA, Gleissner CA (2011). Expression of IL-17A in human atherosclerotic lesions is associated with increased inflammation and plaque vulnerability. Basic Res Cardiol.

[47] Erbel C, Akhavanpoor M, Okuyucu D, Wangler S, Dietz A, Zhao L, Stellos K, Little KM, Lasitschka F, Doesch A, Hakimi M, Dengler TJ, Giese T, Blessing E, Katus HA, Gleissner CA (2014). IL-17A influences essential functions of the monocyte/macrophage lineage and is involved in advanced murine and human atherosclerosis. J Immunol.

[48] Gistera A, Robertson AK, Andersson J, Ketelhuth DF, Ovchinnikova O, Nilsson SK, Lundberg AM, Li MO, Flavell RA, Hansson GK (2013). Transforming growth factor-beta signaling in T cells promotes stabilization of atherosclerotic plaques through an interleukin-17-dependent pathway. Sci Transl Med.

[49] Saigusa R, Winkels H, Ley K (2020). T cell subsets and functions in atherosclerosis. Nat Rev Cardiol.

[50] Lopez-Dee Z, Pidcock K, Gutierrez LS (2011). Thrombospondin-1: multiple paths to inflammation. Mediators Inflamm.

[51] Li Z, He L, Wilson K, Roberts D (2001). Thrombospondin-1 inhibits TCR-mediated T lymphocyte early activation. J Immunol.

[52] Vallejo AN, Mugge LO, Klimiuk PA, Weyand CM, Goronzy JJ (2000). Central role of thrombospondin-1 in the activation and clonal expansion of inflammatory T cells. J Immunol.

[53] Li Q, Li J, Sun L, Sun Y, Zhao F, Liu P, Peng X, Xuan X, Li Y, Wang P, Tan C, Du Y (2021). Exosomes derived from LPS-stimulated human thymic mesenchymal stromal cells enhance inflammation via thrombospondin-1. Biosci Rep.

[54] Boring L, Gosling J, Cleary M, Charo IF (1998). Decreased lesion formation in CCR2-/- mice reveals a role for chemokines in the initiation of atherosclerosis. Nature.

[55] Branen L, Hovgaard L, Nitulescu M, Bengtsson E, Nilsson J, Jovinge S (2004). Inhibition of tumor necrosis factor-alpha reduces atherosclerosis in apolipoprotein E knockout mice. Arterioscler Thromb Vasc Biol.

[56] Ohta H, Wada H, Niwa T, Kirii H, Iwamoto N, Fujii H, Saito K, Sekikawa K, Seishima M (2005). Disruption of tumor necrosis factor-alpha gene diminishes the development of atherosclerosis in ApoE-deficient mice. Atherosclerosis.

[57] Belge KU, Dayyani F, Horelt A, Siedlar M, Frankenberger M, Frankenberger B, Espevik T, Ziegler-Heitbrock L (2002). The proinflammatory CD14+CD16+DR++ monocytes are a major source of TNF. J Immunol.

[58] Fingerle G, Pforte A, Passlick B, Blumenstein M, Strobel M, Ziegler-Heitbrock HW. The novel subset of CD14+/CD16+ blood monocytes is expanded in sepsis patients. Blood. 1993, https://www.ncbi.nlm.nih.gov/pubmed/7693040. 7693040

[59] Castano D, Garcia LF, Rojas M (2011). Increased frequency and cell death of CD16+ monocytes with Mycobacterium tuberculosis infection. Tuberculosis (Edinb).

[60] Soares G, Barral A, Costa JM, Barral-Netto M, Van Weyenbergh J (2006). CD16+ monocytes in human cutaneous leishmaniasis: increased ex vivo levels and correlation with clinical data. J Leukoc Biol.

[61] Kawanaka N, Yamamura M, Aita T, Morita Y, Okamoto A, Kawashima M, Iwahashi M, Ueno A, Ohmoto Y, Makino H (2002). CD14+, CD16+ blood monocytes and joint inflammation in rheumatoid arthritis. Arthritis Rheum.

[62] Nockher WA, Scherberich JE (1998). Expanded CD14+ CD16+ monocyte subpopulation in patients with acute and chronic infections undergoing hemodialysis. Infect Immun.

[63] Sadeghi HM, Schnelle JF, Thoma JK, Nishanian P, Fahey JL (1999). Phenotypic and functional characteristics of circulating monocytes of elderly persons. Exp Gerontol.

[64] Seidler S, Zimmermann HW, Bartneck M, Trautwein C, Tacke F (2010). Age-dependent alterations of monocyte subsets and monocyte-related chemokine pathways in healthy adults. BMC Immunol.

[65] Hearps AC, Martin GE, Angelovich TA, Cheng WJ, Maisa A, Landay AL, Jaworowski A, Crowe SM (2012). Aging is associated with chronic innate immune activation and dysregulation of monocyte phenotype and function. Aging Cell.

[66] Idzkowska E, Eljaszewicz A, Miklasz P, Musial WJ, Tycinska AM, Moniuszko M (2015). The role of different monocyte subsets in the pathogenesis of atherosclerosis and acute coronary syndromes. Scand J Immunol.

[67] Schlitt A, Heine GH, Blankenberg S, Espinola-Klein C, Dopheide JF, Bickel C, Lackner KJ, Iz M, Meyer J, Darius H, Rupprecht HJ (2004). CD14+CD16+ monocytes in coronary artery disease and their relationship to serum TNF-alpha levels. Thromb Haemost.

[68] Kashiwagi M, Imanishi T, Tsujioka H, Ikejima H, Kuroi A, Ozaki Y, Ishibashi K, Komukai K, Tanimoto T, Ino Y, Kitabata H, Hirata K, Akasaka T (2010). Association of monocyte subsets with vulnerability characteristics of coronary plaques as assessed by 64-slice multidetector computed tomography in patients with stable angina pectoris. Atherosclerosis.

[69] Rogacev KS, Cremers B, Zawada AM, Seiler S, Binder N, Ege P, Grosse-Dunker G, Heisel I, Hornof F, Jeken J, Rebling NM, Ulrich C, Scheller B, Bohm M, Fliser D, Heine GH (2012). CD14++CD16+ monocytes independently predict cardiovascular events: a cohort study of 951 patients referred for elective coronary angiography. J Am Coll Cardiol.

[70] Kapellos TS, Bonaguro L, Gemund I, Reusch N, Saglam A, Hinkley ER, Schultze JL (2019). Human monocyte subsets and phenotypes in major chronic inflammatory diseases. Front Immunol.

[71] Uryga AK, Grootaert MOJ, Garrido AM, Oc S, Foote K, Chappell J, Finigan A, Rossiello F, d'Adda di Fagagna F, Aravani D, Jorgensen HF, Bennett MR. Telomere damage promotes vascular smooth muscle cell senescence and immune cell recruitment after vessel injury. Commun Biol. 2021; 10.1038/s42003-021-02123-z. 10.1038/s42003-021-02123-z PMC8140103 34021256

