# Supplementary material for: Unbiased proteomic analysis of extracellular vesicles secreted by senescent human vascular smooth muscle cells reveals their ability to modulate immune cell functions
Source: GeroScience. 2022 Jul 28;44(6):2863–84. doi: 10.1007/s11357-022-00625-0 (PMC9768090; doi:10.1007/s11357-022-00625-0)
Supplement: Supplementary file 1 — Supplementary file1 (PDF 1555 KB) [file 11357_2022_625_MOESM1_ESM.pdf]

Supplementary data

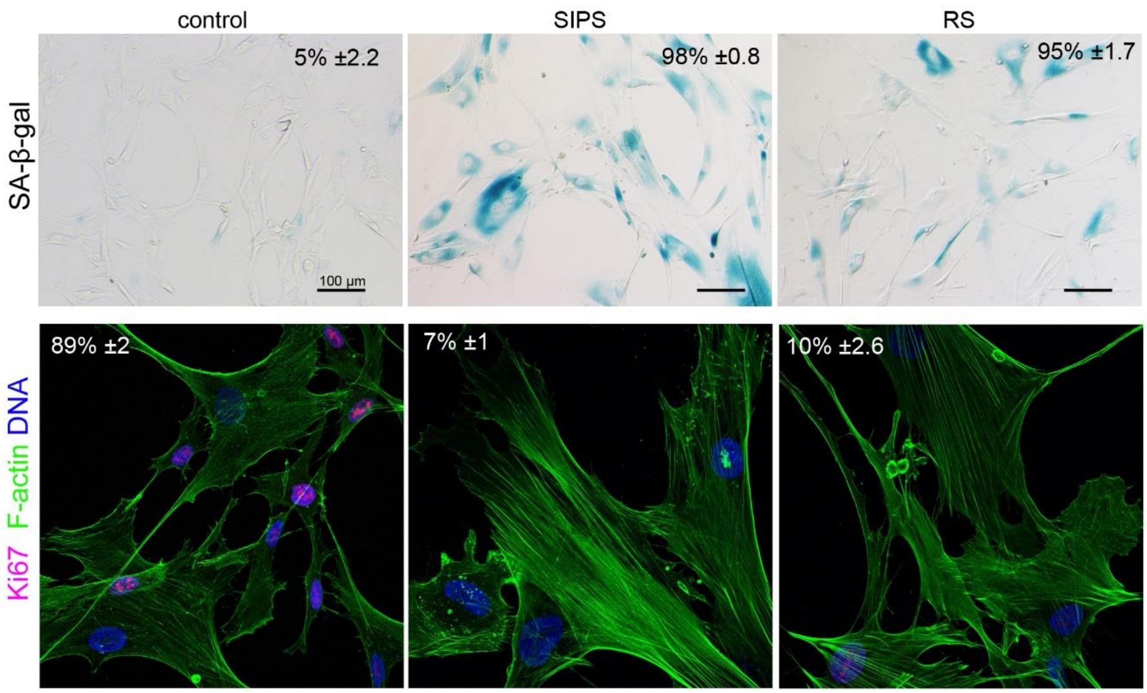

Figure S1. *Characterization of senescent VSMCs.*

Analysis of SA-β-gal activity in young cells (control), H2O2-treated cells (SIPS) and cells undergoing replicative senescence (RS);control refers to untreated cell on an early passage Immunocytochemical detection of proliferation marker Ki-67 in control and senescent VSMCs

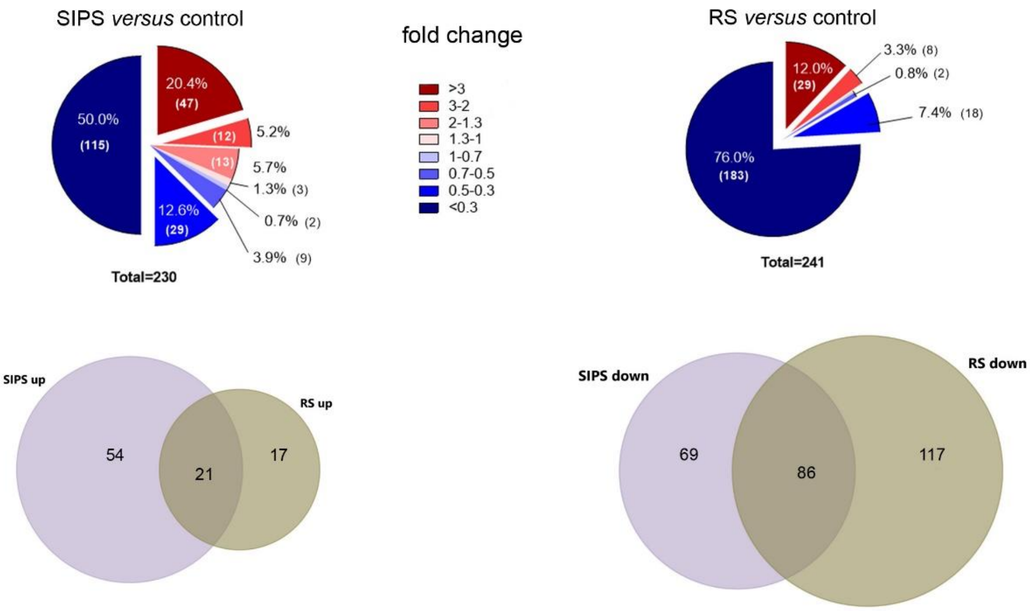

Figure S2. *Proteomic analysis of soluble components of SASP (sSASP) secreted by young and senescent VSMC*

a. Pie charts presenting the distribution of fold changes in significantly changed proteins of EVs secreted by senescent cells relative to EVs from control cells. B.. Venn diagrams of up- and downregulated proteins identified in EVs from RS and SIPS cells that were significantly changed (p<0.05)

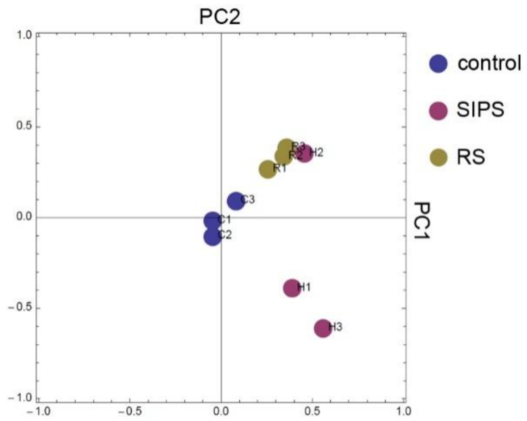

Figure S3 *Principal component analysis (PCA) of protein composition of EVs.*

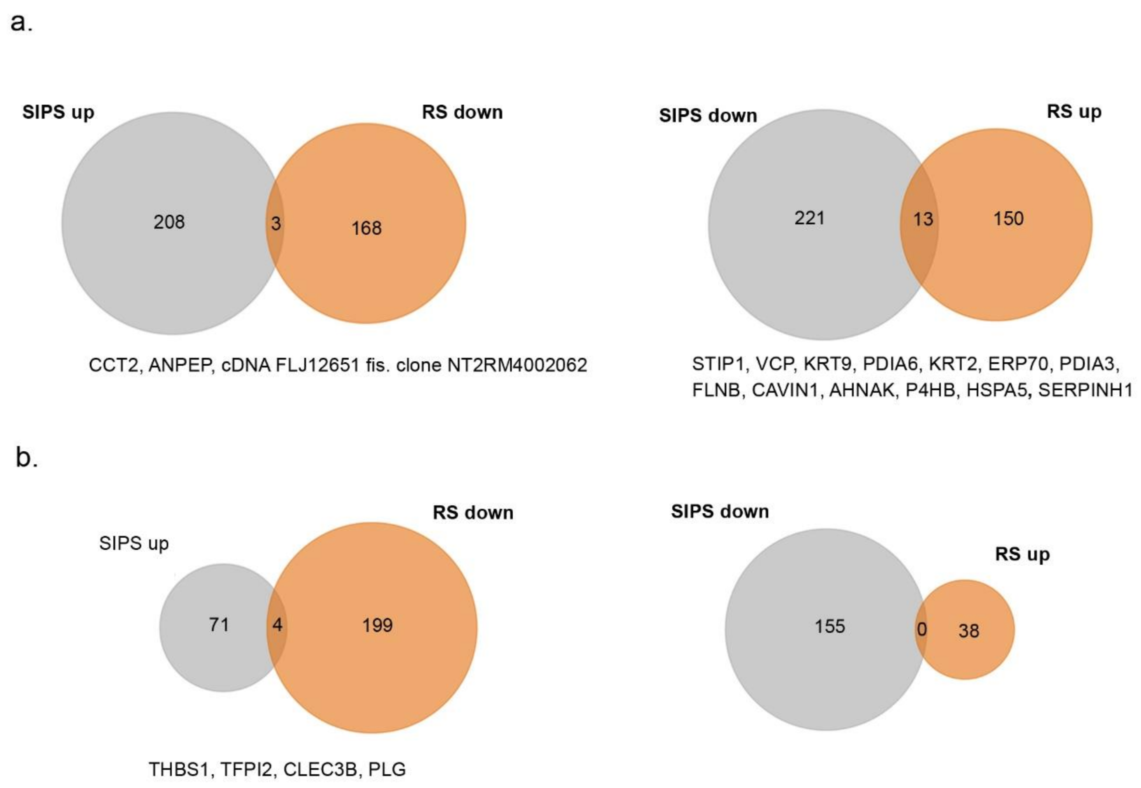

Figure S4. *Venn diagrams presenting proteins identified in EVs (a.) and sSASP (b.) that changed inversely in the secretome of VSMCs undergoing SIPS and RS comparing to control. The proteins identified in both SIPS and RS secretomes are listed below each Venn diagram.*

a.

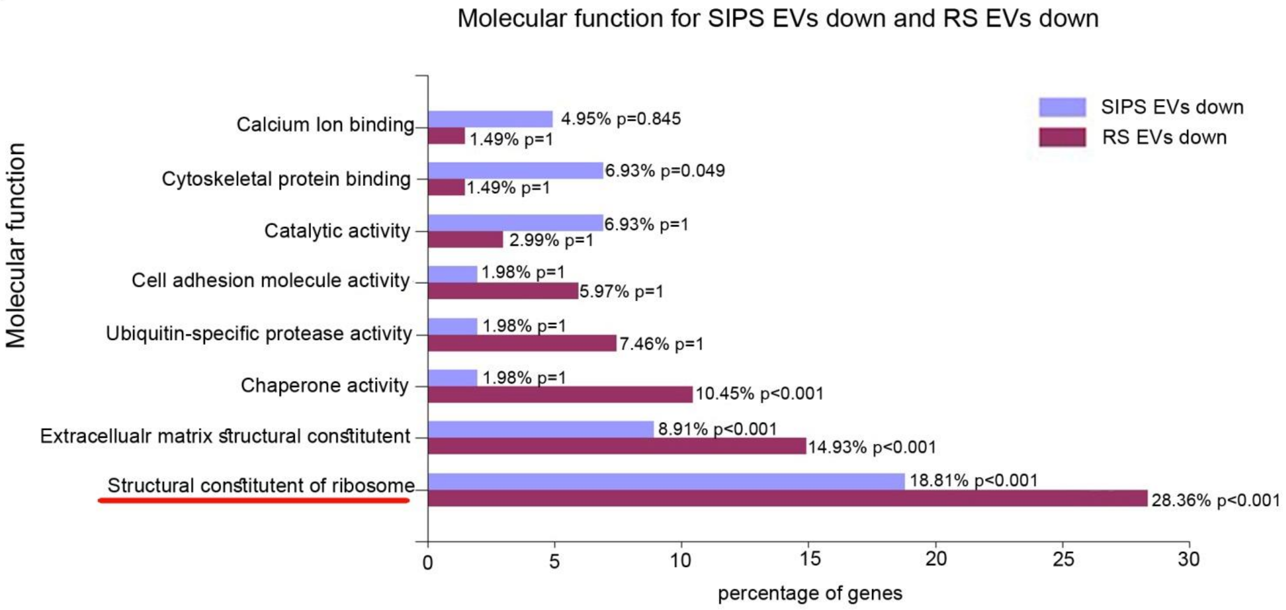

b.

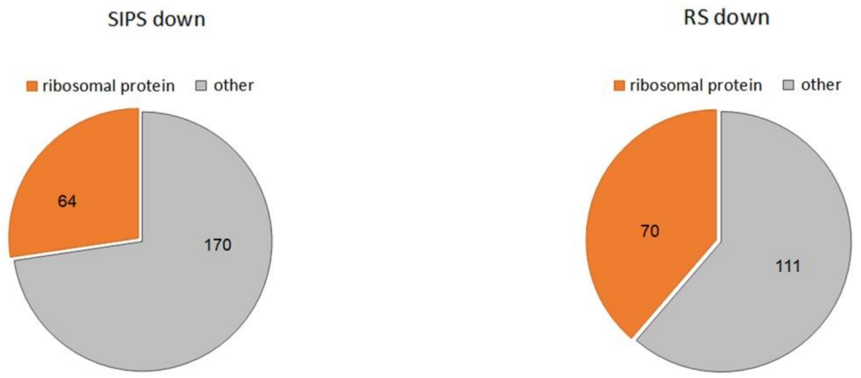

Figure S5. *Ribosomal proteins as the most highly represented group of downregulated proteins identified in senEVs. a. Molecular function pathway analysis of proteins which were significantly downregulated (p<0.05) in senEVs (SIPS and RS) comparing to EVs secreted by young VSMCs; b. Venn diagrams presenting quantitative share of ribosomal proteins among all identified proteins which were downregulated in senEVs*

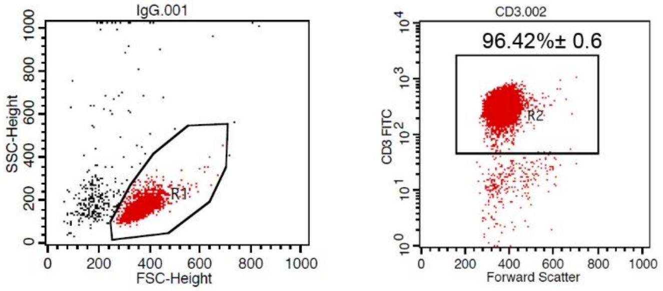

Figure S6. *Estimation of the purity of CD3+ fraction obtained after isolation.*

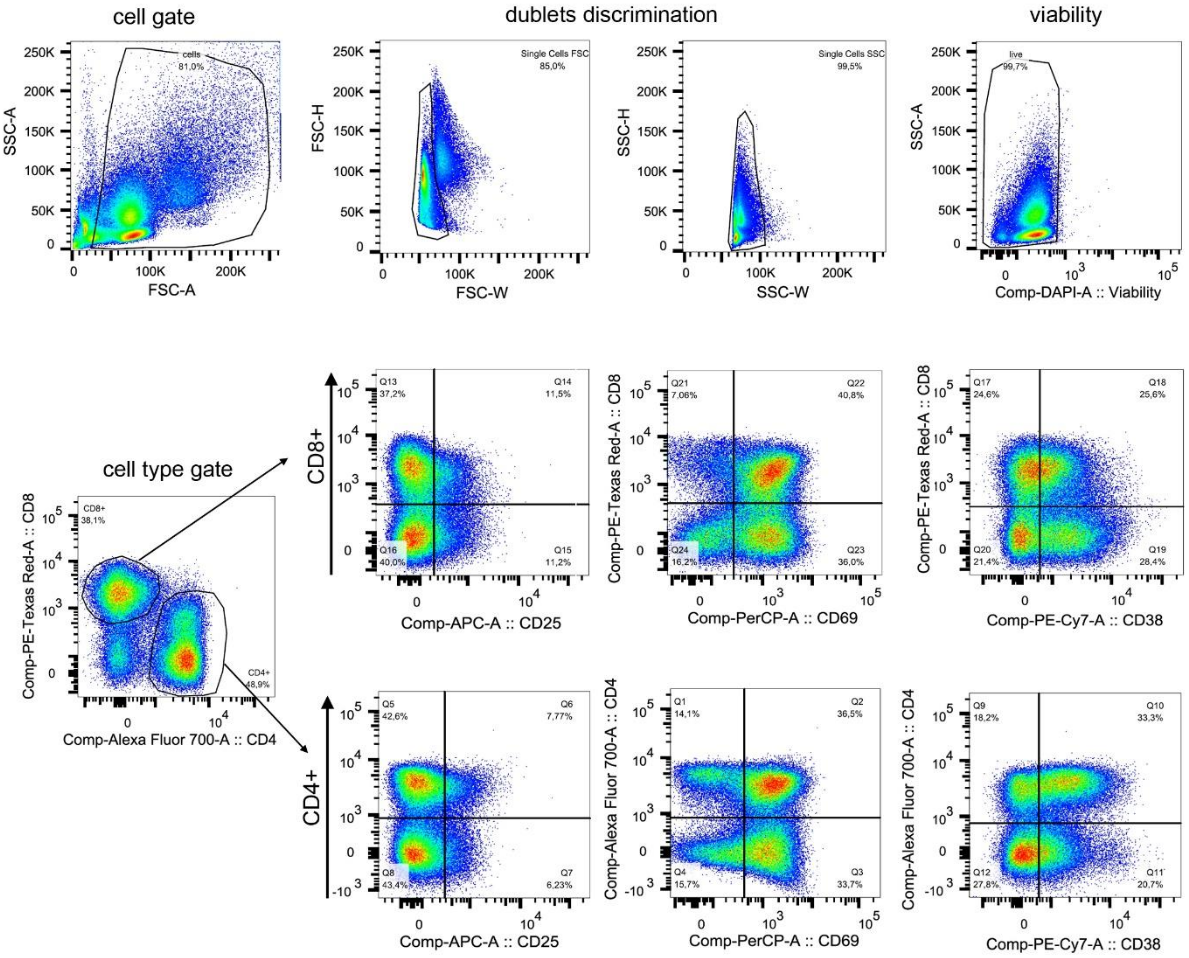

Figure S7. *Representative dot blot showing gating strategy used for estimation of expression of CD25, CD69 and CD38 in CD3+ cells after beads activation.*

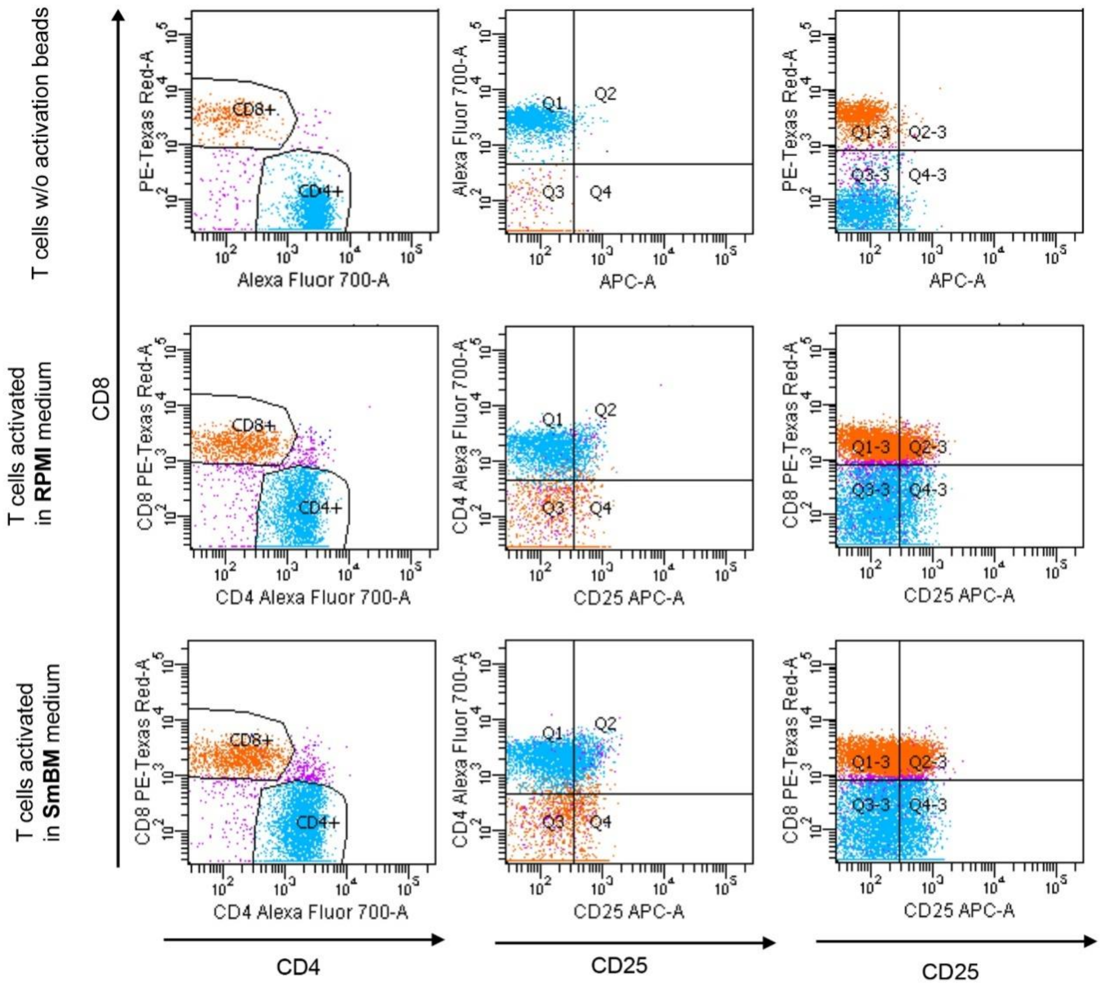

	CD4+/CD25+	CD4+/CD69+	CD4+/CD38+	CD8+/CD25+	CD8+/CD69+	CD8+/CD38+
Without beads	0.1%	0%	2.2%	0.2%	0.2%	0.7%
RPMI medium	12%	30.4%	8.2%	8%	9.1%	2.4%
SmBM medium	17.1%	35.7%	9.7%	11.8%	13%	3.2%

Figure S8. *Representative dot blots presenting the level of expression of CD25 in CD3+ cells in cell cultured without activation beads and activated in different cell culture medium (RPMI and SmBM). Below the exact percentage of particular T cells subpopulation is shown.*

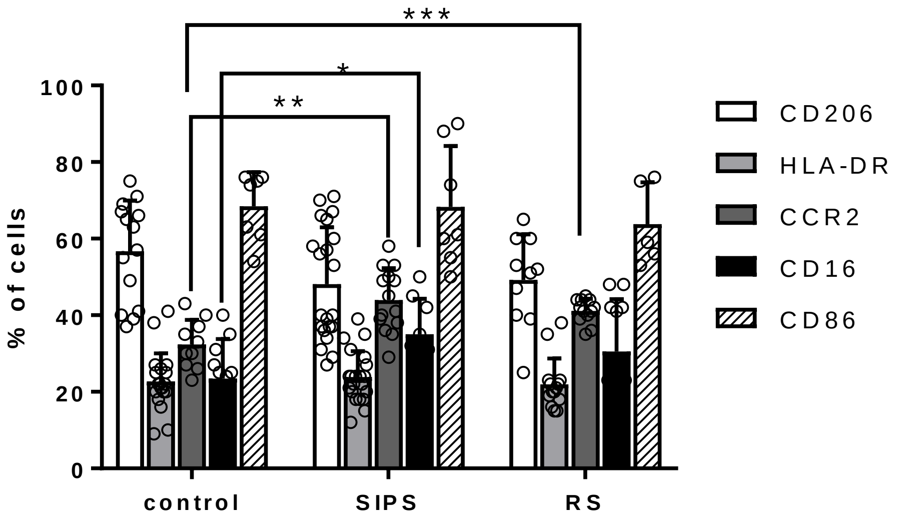

Figure S9. *EVs secreted by senescent VSMCs influenced monocyte differentiation*

Blood derived monocytes were cultured for 7 days in the presence of EVs secreted by control and senescent (SIPS and RS) VSMCs in order to differentiate into monocyte derived macrophages (MDM). Thereafter the polarization of MDM were determined using selected markers. Statistical analysis was performed using t-test.

Table S1 *Proteins common for SIPS and RS EVs, which increased at least 2 times comparing to control or changed less (fold change >1.3) but with the highest significance (p<0.001)* ***(bold font)***

EVs TOP11 (H2O2 + RS)

identified proteins	protein ID	Fold change log_2 SIPS	Fold change log_2 RS
cDNA FLJ55489	RIPOR3	19,08	17,8
Hydroxysteroid dehydrogenase 10 isoform 1	HSD17B10	2,41	2,49
Lipoma HMGIC fusion partner-like 2	LHFPL2	7,74	2,09
cDNA. FLJ93871. highly similar melanoma antigen. family B	MAGEB2	1,70	1,36
**cDNA FLJ51509. highly similar to Alpha-fetoprotein**	**AFP**	**1,45**	**1,41**
**Protein-glutamine gamma-glutamyltransferase 2**	**TGM2**	**1,41**	**1,07**
Serpin peptidase inhibitor. clade C (Antithrombin)	SERPINC1	**1,32**	0,73
Cytoskeleton-associated protein 4.	CKAP4	1,00	**0,87**
**Keratin. type I cytoskeletal 16**	**KRT16**	**0,47**	**1,09**
**Vimentin**	**VIM**	**0,64**	**0,56**
**Heat shock protein beta-1**	**HSPB1**	**0,63**	**0,55**

Table S2 *Proteins unique for SIPS EVs, which increased at least 2 times comparing to control or changed less (fold change >1.3) but with the highest significance (p<0.001)* ***(bold font)***

EVs TOP14 (H_2O_2 only)

identified proteins	protein ID	Fold change log_2
NAD-dependent protein deacetylase sirtuin-3	SIRT3	8,78
Protein Wnt	WNT5A	3,34
CXADR-like membrane protein	CLMP	1,4
**highly similar to cartilage oligomeric matrix protein (COMP)**	**COMP**	**1,32**
Dynamin-2	DNM2	1,18
Vacuolar protein sorting 37B	VPS37B	1,08
Uncharacterized protein KIAA1683	KIAA1683	1,07
**Metalloendopeptidase**	**BMP1**	**0,9**
**Prostaglandin F2 receptor negative regulator**	**PTGFRN**	**0,89**
**Protocadherin Fat 1**	**FAT1**	**0,85**
**Inter-alpha (Globulin) inhibitor H2**	**ITIH2**	**0,84**
**EH-domain containing 4. isoform CRA_a**	**EHD4**	**0,47**
**EH-domain containing 2. isoform CRA_a**	**EHD2**	**0,46**
**Kinesin light chain 1**	**KLC1**	**0,39**

Table S3 *Proteins unique for RS EVs, which increased at least 2 times comparing to control or changed less (fold change >1.3) but with the highest significance (p<0.001)* ***(bold font)***

EVs TOP13 (RS only)

identified proteins	protein ID	Fold change log_2
Arginase-1	ARG1	2,27
Truncated profilaggrin	FLG	1,31
Thioredoxin domain-containing protein 5	TXNDC5	1,01
Splicing factor. arginine/serine-rich 2.	SFRS2	0,98
highly similar to receptor alpha (translocon-associated protein alpha) (SSR1).	SSR1	0,98
Superoxide dismutase	SOD2	0,98
Catalase	CAT	0,98
Splicing factor. arginine/serine-rich 2	SFRS2	0,98
**Endoplasmin**	**HSP90B1**	**0,75**
**Protein disulfide-isomerase A3**	**PDIA3**	**0,71**
**78 kDa glucose-regulated protein**	**HSPA5**	**0,6**
**Phosphoglycerate kinase 1**	**PGK1**	**0,55**
**Transitional endoplasmic reticulum ATPase**	**VCP**	**0,38**

Table S4 *Proteins common for SIPS and RS sSASP, which increased at least 2 times comparing to control or changed less (fold change >1.3) but with the highest significance (p<0.001)* ***(bold font)***

sSASP TOP10 (H_2O_2 + RS)

identified proteins	protein ID	Fold change log_2 SIPS	Fold change log_2 RS
Epididymis secretory sperm binding protein Li 71p	HEL-S-71p	5,1	4,05
Tumor necrosis factor receptor superfamily member 12A	TNFRSF12A	2,34	4,78
Complement factor I	CFI	2,79	3,43
Ubiquitin carboxyl-terminal hydrolase	UCHL5	2,5	3,21
Serine/threonine-protein phosphatase 2A activator	B2RAN2	2,08	2,23
moderately similar to Beta-2-glycoprotein 1	APOH	1,95	2,37
highly similar to vanin 1 (VNN1)	PTPA	1,53	2,51
Glypican-1	GPC1	1,47	**2,19**
Fructose-2.6-bisphosphatase TIGAR	ALDOC	1,06	**2,13**
Granulocyte macrophage-colony stimulating factor	CSF2	0,78	1,55

Table S5 *Proteins unique for SIPS sSASP, which increased at least 2 times comparing to control or changed less (fold change >1.3) but with the highest significance (p<0.001)* ***(bold font)***

sSASP TOP12 (H_2O_2 only)

identified proteins	protein ID	Fold change log_2
**Lactoferrin**	**LTF**	**4,15**
Prothrombin	F2	2,85
**Vitamin D-binding protein**	**GC**	**2,68**
highly similar to Human complement protein component C7	A8K2T4	2,56
**Connective tissue growth factor**	**CTGF**	**2,2**
Inter-alpha (Globulin) inhibitor H4 (Plasma Kallikrein-sensitive glycoprotein)	ITIH4	2,1
Thyroxine-binding globulin	SERPINA7	2,07
**Protein-glutamine gamma-glutamyltransferase 2**	**TGM2**	**1,99**
Purine nucleoside phosphorylase	PNP	1,97
highly similar to Alpha-fetoprotein	AFP	1,86
C-type lectin domain family 3. member B. isoform CRA	CLEC3B	1,84
Tissue factor pathway inhibitor 2	TFPI2	1,6

Table S6 *Proteins unique for RS sSASP, which increased at least 2 times comparing to control or changed less (fold change >1.3) but with the highest significance (p<0.001)* ***(bold font)***

sSASP TOP13 (RS only)

identified proteins	protein ID	Fold change log_2
Rho GTPase-activating protein 1	ARHGAP1	4,03
Lumican	LUM	2,78
Macrophage colony-stimulating factor 1	CSF1	2,49
Testicular secretory protein Li 65	ATP6V1B2	2,4
Protein phosphatase 2 regulatory subunit A (PR 65). alpha	PPP2R1A	2,28
**Sushi. von Willebrand factor type A. EGF and pentraxin domain-containing protein 1**	**SVEP1**	**2,05**
Ubiquitin-conjugating enzyme E2L 3	UBE2L3	2,04
Crk-like protein	CRKL	1,99
SEC31-like 1 (S. cerevisiae	SEC31L1	1,85
Uncharacterized protein (Fragment)	F8W031	1,7
