# Supplementary material for: Unbiased proteomic analysis of extracellular vesicles secreted by senescent human vascular smooth muscle cells reveals their ability to modulate immune cell functions
Source: GeroScience. 2022 Jul 28;44(6):2863–84. doi: 10.1007/s11357-022-00625-0 (PMC9768090; doi:10.1007/s11357-022-00625-0)
Supplement: Supplementary file 2 — Supplementary file2 (PDF 237 KB) [file 11357_2022_625_MOESM2_ESM.pdf]

Table Supplementary 7 ClueGO Gene Ontology Biological Process SIPS up EVs

Gene Ontology Pathways (ID/term)		Proteins
GO:0002263	cell activation involved in immune response	[ALDOC, ANPEP, C3, CAPN1, CCT2, CKAP4, CLU, CTSB, DSC1, DYNC1H1, DYNC1LI1, FTH1, FTL, GLB1, GSN, HBB, LRP1, MME, PFKL, PGM1, PKM, PSMC2, PSMD1, PSMD12, PTX3, PXDN, PYGL, RAB6A, SNX6, TUBB, VAT1, XRCC6]
GO:0002274	myeloid leukocyte activation	[ALDOC, ANPEP, C3, CAPN1, CCT2, CKAP4, CLU, CTSB, DSC1, DYNC1H1, DYNC1LI1, FTH1, FTL, GLB1, GSN, HBB, MME, NECTIN2, PFKL, PGM1, PKM, PSMC2, PSMD1, PSMD12, PTX3, PXDN, PYGL, RAB6A, SNX6, THBS1, TUBB, VAT1, WNT5A, XRCC6]
GO:0002444	myeloid leukocyte mediated immunity	[ALDOC, ANPEP, C3, CAPN1, CCT2, CKAP4, CLU, CTSB, DSC1, DYNC1H1, DYNC1LI1, FTH1, FTL, GLB1, GSN, HBB, MME, PFKL, PGM1, PKM, PSMC2, PSMD1, PSMD12, PTX3, PXDN, PYGL, RAB6A, SNX6, TUBB, VAT1, XRCC6]
GO:0032940	secretion by cell	[A2M, ACTN1, AIMP1, ALDOC, ANPEP, C1QTNF3, C3, CAPN1, CCT2, CKAP4, CLU, COMP, COPS5, CTSB, DPP4, DSC1, DYNC1H1, DYNC1LI1, F5, FGB, FLNA, FTH1, FTL, GAS6, GLB1, GSN, HBB, ITGB3, KIF5B, LHFPL2, LRP1, MME, NSF, PDCD6IP, PFKL, PFKM, PGM1, PKM, PLG, PSMC2, PSMD1, PSMD12, PTX3, PXDN, PYGL, RAB6A, SEPTIN2, SERPINE1, SIRT3, SNX6, SPARC, THBS1, TLN1, TSG101, TUBA4A, TUBB, VAT1, XRCC6]
GO:0002275	myeloid cell activation involved in immune response	[ALDOC, ANPEP, C3, CAPN1, CCT2, CKAP4, CLU, CTSB, DSC1, DYNC1H1, DYNC1LI1, FTH1, FTL, GLB1, GSN, HBB, MME, PFKL, PGM1, PKM, PSMC2, PSMD1, PSMD12, PTX3, PXDN, PYGL, RAB6A, SNX6, TUBB, VAT1, XRCC6]
GO:0006887	exocytosis	[A2M, ACTN1, ALDOC, ANPEP, C3, CAPN1, CCT2, CKAP4, CLU, COPS5, CTSB, DSC1, DYNC1H1, DYNC1LI1, F5, FGB, FLNA, FTH1, FTL, GAS6, GLB1, GSN, HBB, ITGB3, LHFPL2, MME, NSF, PDCD6IP, PFKL, PGM1, PKM, PLG, PSMC2, PSMD1, PSMD12, PTX3, PXDN, PYGL, RAB6A, SEPTIN2, SERPINE1, SNX6, SPARC, THBS1, TLN1, TSG101, TUBA4A, TUBB, VAT1, XRCC6]
GO:0036230	granulocyte activation	[ALDOC, ANPEP, C3, CAPN1, CCT2, CKAP4, CLU, CTSB, DSC1, DYNC1H1, DYNC1LI1, FTH1, FTL, GLB1, GSN, HBB, MME, PFKL, PGM1, PKM, PSMC2, PSMD1, PSMD12, PTX3, PXDN, PYGL, RAB6A, TUBB, VAT1, XRCC6]
GO:0002446	neutrophil mediated immunity	[ALDOC, ANPEP, C3, CAPN1, CCT2, CKAP4, CLU, CTSB, DSC1, DYNC1H1, DYNC1LI1, FTH1, FTL, GLB1, GSN, HBB, MME, PFKL, PGM1, PKM, PSMC2, PSMD1, PSMD12, PTX3, PXDN, PYGL, RAB6A, TUBB, VAT1, XRCC6]
GO:0042119	neutrophil activation	[ALDOC, ANPEP, C3, CAPN1, CCT2, CKAP4, CLU, CTSB, DSC1, DYNC1H1, DYNC1LI1, FTH1, FTL, GLB1, GSN, HBB, MME, PFKL, PGM1, PKM, PSMC2, PSMD1, PSMD12, PTX3, PXDN, PYGL, RAB6A, TUBB, VAT1, XRCC6]
GO:0043299	leukocyte degranulation	[ALDOC, ANPEP, C3, CAPN1, CCT2, CKAP4, CLU, CTSB, DSC1, DYNC1H1, DYNC1LI1, FTH1, FTL, GLB1, GSN, HBB, MME, PFKL, PGM1, PKM, PSMC2, PSMD1, PSMD12, PTX3, PXDN, PYGL, RAB6A, SNX6, TUBB, VAT1, XRCC6]
GO:0045055	regulated exocytosis	[A2M, ACTN1, ALDOC, ANPEP, C3, CAPN1, CCT2, CKAP4, CLU, CTSB, DSC1, DYNC1H1, DYNC1LI1, F5, FGB, FLNA, FTH1, FTL, GAS6, GLB1, GSN, HBB, ITGB3, LHFPL2, MME, PFKL, PGM1, PKM, PLG, PSMC2, PSMD1, PSMD12, PTX3, PXDN, PYGL, RAB6A, SERPINE1, SNX6, SPARC, THBS1, TLN1, TUBA4A, TUBB, VAT1, XRCC6]
GO:0002283	neutrophil activation involved in immune response	[ALDOC, ANPEP, C3, CAPN1, CCT2, CKAP4, CLU, CTSB, DSC1, DYNC1H1, DYNC1LI1, FTH1, FTL, GLB1, GSN, HBB, MME, PFKL, PGM1, PKM, PSMC2, PSMD1, PSMD12, PTX3, PXDN, PYGL, RAB6A, TUBB, VAT1, XRCC6]
GO:0002576	platelet degranulation	[A2M, ACTN1, CLU, F5, FGB, FLNA, GAS6, ITGB3, LHFPL2, PLG, SERPINE1, SPARC, THBS1, TLN1, TUBA4A]
GO:0043312	neutrophil degranulation	[ALDOC, ANPEP, C3, CAPN1, CCT2, CKAP4, CLU, CTSB, DSC1, DYNC1H1, DYNC1LI1, FTH1, FTL, GLB1, GSN, HBB, MME, PFKL, PGM1, PKM, PSMC2, PSMD1, PSMD12, PTX3, PXDN, PYGL, RAB6A, TUBB, VAT1, XRCC6]
GO:0002504	antigen processing and presentation of peptide or polysaccharide antigen via MHC class II	[ACTR1A, AP2B1, CLTC, DCTN1, DCTN2, DNM2, DYNC1H1, DYNC1LI1, DYNC1LI2, KLC1, SEC31A, THBS1]

GO:0019884 antigen processing and presentation of exogenous antigen	[ACTR1A, AP2B1, CLTC, DCTN1, DCTN2, DNM2, DYNC1H1, DYNC1LI1, DYNC1LI2, KLC1, PSMB8, PSMC2, PSMC5, PSMD1, PSMD12, SEC31A]
GO:0042180 cellular ketone metabolic process	[AFP, AKR1B1, APOA4, APOC3, HSD17B10, NQO1, PSMB8, PSMC2, PSMC5, PSMD1, PSMD12]
GO:0048002 antigen processing and presentation of peptide antigen	[ACTR1A, AP2B1, CLTC, DCTN1, DCTN2, DNM2, DYNC1H1, DYNC1LI1, DYNC1LI2, KLC1, PSMB8, PSMC2, PSMC5, PSMD1, PSMD12, SEC31A]
GO:0007164 establishment of tissue polarity	[AP2B1, CLTC, CTHRC1, PSMB8, PSMC2, PSMC5, PSMD1, PSMD12, WNT5A]
GO:0140056 organelle localization by membrane tethering	[ACTR1A, DCTN1, DCTN2, DYNC1H1, MAPRE1, NSF, TUBA1A, TUBA4A, TUBB, USO1]
GO:0002478 antigen processing and presentation of exogenous peptide antigen	[ACTR1A, AP2B1, CLTC, DCTN1, DCTN2, DNM2, DYNC1H1, DYNC1LI1, DYNC1LI2, KLC1, PSMB8, PSMC2, PSMC5, PSMD1, PSMD12, SEC31A]
GO:0002495 antigen processing and presentation of peptide antigen via MHC class II	[ACTR1A, AP2B1, CLTC, DCTN1, DCTN2, DNM2, DYNC1H1, DYNC1LI1, DYNC1LI2, KLC1, SEC31A]
GO:0032103 positive regulation of response to external stimulus	[C3, CDH13, FLNA, GAS6, HSPB1, LRP1, NECTIN2, OXSR1, PDGFRB, PLG, PSMB8, PSMC2, PSMC5, PSMD1, PSMD12, SERPINE1, TGM2, THBS1, THBS4, TPBG, WNT5A, XRCC6]
GO:0048193 Golgi vesicle transport	[ACTR1A, CLTC, COPZ1, DCTN1, DCTN2, DNM2, DYNC1H1, DYNC1LI1, DYNC1LI2, F5, FTH1, FTL, GAS6, KLC1, NSF, RAB6A, SEC31A, SNX3, TFG, USO1]
GO:0006520 cellular amino acid metabolic process	[AHCY, AIMP1, GARS1, GFPT1, GOLT1B, HSD17B10, IARS1, MTHFD1, NQO1, PSMB8, PSMC2, PSMC5, PSMD1, PSMD12, VARS1]
GO:0044839 cell cycle G2/M phase transition	[ACTR1A, DCTN1, DCTN2, DNM2, DYNC1H1, MAPRE1, PPP1CB, PSMB8, PSMC2, PSMC5, PSMD1, PSMD12, TUBA1A, TUBA4A, TUBB]
GO:0006888 endoplasmic reticulum to Golgi vesicle-mediated transport	[ACTR1A, COPZ1, DCTN1, DCTN2, DYNC1H1, DYNC1LI1, DYNC1LI2, F5, GAS6, NSF, SEC31A, TFG, USO1]
GO:0019886 antigen processing and presentation of exogenous peptide antigen via MHC class II	[ACTR1A, AP2B1, CLTC, DCTN1, DCTN2, DNM2, DYNC1H1, DYNC1LI1, DYNC1LI2, KLC1, SEC31A]
GO:0038061 NIK/NF-kappaB signaling	[C1QTNF3, GREM1, ILK, PSMB8, PSMC2, PSMC5, PSMD1, PSMD12, TNFRSF10B]
GO:0000086 G2/M transition of mitotic cell cycle	[ACTR1A, DCTN1, DCTN2, DNM2, DYNC1H1, MAPRE1, PPP1CB, PSMB8, PSMC2, PSMC5, PSMD1, PSMD12, TUBA1A, TUBA4A, TUBB]
GO:0001736 establishment of planar polarity	[AP2B1, CLTC, CTHRC1, PSMB8, PSMC2, PSMC5, PSMD1, PSMD12, WNT5A]
GO:0001738 morphogenesis of a polarized epithelium	[AP2B1, CLTC, CTHRC1, FAT1, PSMB8, PSMC2, PSMC5, PSMD1, PSMD12, WNT5A]
GO:0006521 regulation of cellular amino acid metabolic process	[NQO1, PSMB8, PSMC2, PSMC5, PSMD1, PSMD12]
GO:2000027 regulation of animal organ morphogenesis	[AKR1B1, AP2B1, CLTC, CTHRC1, GREM1, PSMB8, PSMC2, PSMC5, PSMD1, PSMD12, TNFRSF11B, WNT5A]
GO:0097711 ciliary basal body-plasma membrane docking	[ACTR1A, DCTN1, DCTN2, DYNC1H1, MAPRE1, TUBA1A, TUBA4A, TUBB]
GO:1902749 regulation of cell cycle G2/M phase transition	[ACTR1A, DCTN1, DCTN2, DYNC1H1, MAPRE1, PSMB8, PSMC2, PSMC5, PSMD1, PSMD12, TUBA1A, TUBA4A, TUBB]

GO:0030178	negative regulation of Wnt signaling pathway	[CTHRC1, GREM1, LRP1, PSMB8, PSMC2, PSMC5, PSMD1, PSMD12, RUVBL2, TPBG, WNT5A]
GO:0035567	non-canonical Wnt signaling pathway	[AP2B1, CLTC, CTHRC1, PSMB8, PSMC2, PSMC5, PSMD1, PSMD12, WNT5A]
GO:0090175	regulation of establishment of planar polarity	[AP2B1, CLTC, CTHRC1, PSMB8, PSMC2, PSMC5, PSMD1, PSMD12, WNT5A]
GO:0010389	regulation of G2/M transition of mitotic cell cycle	[ACTR1A, DCTN1, DCTN2, DYNC1H1, MAPRE1, PSMB8, PSMC2, PSMC5, PSMD1, PSMD12, TUBA1A, TUBA4A, TUBB]
GO:0060071 pathway	Wnt signaling pathway, planar cell polarity	[AP2B1, CLTC, CTHRC1, PSMB8, PSMC2, PSMC5, PSMD1, PSMD12, WNT5A]
GO:0090090 pathway	negative regulation of canonical Wnt signaling	[CTHRC1, GREM1, PSMB8, PSMC2, PSMC5, PSMD1, PSMD12, RUVBL2, TPBG, WNT5A]
