# Supplementary material for: Unbiased proteomic analysis of extracellular vesicles secreted by senescent human vascular smooth muscle cells reveals their ability to modulate immune cell functions
Source: GeroScience. 2022 Jul 28;44(6):2863–84. doi: 10.1007/s11357-022-00625-0 (PMC9768090; doi:10.1007/s11357-022-00625-0)
Supplement: Supplementary file 3 — Supplementary file3 (PDF 223 KB) [file 11357_2022_625_MOESM3_ESM.pdf]

Table Supplementary 8 ClueGO Gene Ontology Biological Process RS up EVs.

Gene Ontology Pathways (ID/term)	Proteins
GO:0098760 response to interleukin-7	[P4HB, PDIA3, RAD23B, STIP1]
GO:0098761 cellular response to interleukin-7	[P4HB, PDIA3, RAD23B, STIP1]
GO:0070671 response to interleukin-12	[HNRNPA2B1, HSPA9, P4HB, SOD2, TALDO1]
GO:0035722 interleukin-12-mediated signaling pathway	[HNRNPA2B1, HSPA9, P4HB, SOD2, TALDO1]
GO:0071349 cellular response to interleukin-12	[HNRNPA2B1, HSPA9, P4HB, SOD2, TALDO1]
GO:0002504 antigen processing and presentation of peptide or polysaccharide antigen via MHC class II	[ACTR1A, CANX, CLTC, CTSD, DCTN1, DYNC1H1, DYNC1LI2, THBS1]
GO:0019884 antigen processing and presentation of exogenous antigen	[ACTR1A, CALR, CANX, CLTC, CTSD, DCTN1, DYNC1H1, DYNC1LI2, PDIA3, PSMD4, SEC22B]
GO:0048002 antigen processing and presentation of peptide antigen	[ACTR1A, CALR, CANX, CLTC, CTSD, DCTN1, DYNC1H1, DYNC1LI2, PDIA3, PSMD4, SEC22B]
GO:0002478 antigen processing and presentation of exogenous peptide antigen	[ACTR1A, CALR, CANX, CLTC, CTSD, DCTN1, DYNC1H1, DYNC1LI2, PDIA3, PSMD4, SEC22B]
GO:0002495 antigen processing and presentation of peptide antigen via MHC class II	[ACTR1A, CANX, CLTC, CTSD, DCTN1, DYNC1H1, DYNC1LI2]
GO:0019886 antigen processing and presentation of exogenous peptide antigen via MHC class II	[ACTR1A, CANX, CLTC, CTSD, DCTN1, DYNC1H1, DYNC1LI2]
