# Supplementary material for: Unbiased proteomic analysis of extracellular vesicles secreted by senescent human vascular smooth muscle cells reveals their ability to modulate immune cell functions
Source: GeroScience. 2022 Jul 28;44(6):2863–84. doi: 10.1007/s11357-022-00625-0 (PMC9768090; doi:10.1007/s11357-022-00625-0)
Supplement: Supplementary file 4 — Supplementary file4 (PDF 971 KB) [file 11357_2022_625_MOESM4_ESM.pdf]

Table S9 *Comparison of proteins identified in the secretome (EVs and sSASP) of VSMCs and fibroblasts and epithelial cells (SASP Atlas data); (**bold font** – p < 0.001; ↑ higher level in VSMC)*

H_2O_2 – hydrogen peroxide induced senescence, IR – X radiation induced senescence, RS – replicative senescence,

Proteins common for EVs from H_2O_2 *VSMC*, RS *VSMC*, IR *fibroblasts*

H_2O_2 VSMC – IR fibro		H_2O_2 VSMC– RS VSMC– IR fibro			RS VSMC– IR fibro	
Protein ID	fold change log₂	Protein ID	fold change log₂		Protein ID	fold change log₂
			H_2O_2	RS		
**WNT5A**	3,34 ↑	SERPINF1	2,67 ↑	1,74 ↑	CALR	0,75
**A2M**	**1,81**	**THBS1**	**0,81**	**1,54**	COL4A2	0,75
**FBLN1**	1,2	GPX1	0,46	0,41	**PDIA3**	0,71
**CLU**	0,9	PLOD1	0,4	**0,47**	**P4HB**	0,68
**ITIH2**	0,84	TNC	0,44	0,41	**FKBP10**	0,67
**POSTN**	0,6	LRP1	0,18	**0,3**	**HSPA5**	0,6
**COL12A1**	0,57	**CLTC**	**0,16**	**0,29**		
**PTX3**	0,55					
**PXDN**	0,53					
**ACTN1**	0,53					
**GREM1**	0,41					
**LCAT**	0,36					
**COL1A2**	0,35					
**C1R**	0,34					
**GFPT1**	0,34					
**C1S**	0,33					
**TGFBI**	0,3					
**IARS**	0,2					
**EMILIN1**	0,18					
**RUVBL2**	0,09					

Proteins common for sSASP form H_2O_2 *VSMC*, RS *VSMC*, IR *fibroblasts*, IR *epithelial cells*

H_2O_2 VSMC – IR fibro – IR epi		H_2O_2 VSMC – RS VSMC – IR fibro			H_2O_2 VSMC – RS VSMC – IR epi		
Protein ID	fold change log₂	Protein ID	fold change log₂		Protein ID	fold change log₂	
			H_2O_2	RS		H_2O_2	RS
**GDF15**	0,91	**IGFBP5**	**1,47**	**2,71**	ARSA	2,31 ↑	2,77 ↑
		ALDOC	1,06 -	**2,13 ↑**			

H_2O_2 VSMC – IR fibro		H_2O_2 VSMC – IR epi		RS VSMC – IR fibro		RS VSMC– IR epi	
**Protein ID**	**fold change log_2**	Protein ID	**fold change log_2**	Protein ID	**fold change log_2**	Protein ID	**fold change log_2**
**RBP4**	2,77	CAPNS1	2 ↑	FN1	4,03 ↑	ACTC1	2,78 ↑
**GC**	2,68 ↑	**TGM2**	**1,99 ↑**	PLOD3	1,13	ATP6V1B2	1,85 ↑
**TGFBI**	2,25 ↑	PRDX3	0,96 ↑			PPP2R1A	1,7 ↑
**CTGF**	2,2 ↑						
**CTHRC1**	1,35 ↑						
**GDI1**	0,72						
**THBS1**	0,6						
**TPI1**	0,59						
**VCL**	0,2						
